# Associating gene expressions with curcuminoid biosynthesis in turmeric

**DOI:** 10.1186/s43141-020-00101-2

**Published:** 2020-12-14

**Authors:** Dipendra Kumar Ayer, Kaushal Modha, Vipulkumar Parekh, Ritesh Patel, Gopal Vadodariya, Vinita Ramtekey, Arpit Bhuriya

**Affiliations:** 1 Department of Genetics and Plant Breeding, N. M. College of Agriculture, Navsari Agricultural University, Navsari, Gujarat 396450 India; 2 Department of Basic Science and Humanity, ASPEE College of Horticulture and Forestry, Navsari Agricultural University, Navsari, Gujarat 396450 India

**Keywords:** Association, Curcuminoids, Diketide-CoA synthase, Gene regulation, Multiple curcumin synthases

## Abstract

**Background:**

Biologically important curcuminoids viz curcumin, demethoxycurcumin, and bisdemethoxycurcumin in turmeric rhizome have several health benefits. Pharmaceutical industries synthesize curcuminoids manipulating gene expressions in vitro or in vivo because of their medicinal importance. In this experiment, we studied the gene expressions involved in the curcuminoid biosynthesis pathway in association with curcuminoid yield in turmeric rhizome to study the impact of individual gene expression on curcuminoid biosynthesis.

**Results:**

Gene expressions at the different growth stages of turmeric rhizome were association tested with respective curcuminoid contents. Gene expression patterns of diketide-CoA synthase (*DCS*) and multiple curcumin synthases (*CURS*s) viz curcumin synthase 1 (*CURS1*), curcumin synthase 2 (*CURS2*), and curcumin synthase 3 (*CURS3*) were differentially associated with different curcuminoid contents. Genotype and growth stages showed a significant effect on the gene expressions resulting in a significant impact on curcuminoid balance in turmeric rhizome. *DCS* and *CURS3* expression patterns were similar but distinct from *CURS1* and *CURS2* expression patterns in turmeric rhizome. *DCS* expression had a significant positive and *CURS3* expression had a significant negative association with curcumin, demethoxycurcumin, bisdemethoxycurcumin, and total curcuminoid in turmeric rhizome. *CURS1* expression had a negative association with curcumin whereas *CURS2* expression showed a positive association with demethoxycurcumin.

**Conclusions:**

The effects of *DCS* and *CURS* expressions are not always positive with different curcuminoid contents in turmeric rhizome. *DCS* expression has a positive and *CURS3* expression has a negative association with curcuminoids. *CURS1* and *CURS2* are also associated with curcumin and demethoxycurcumin synthesis. This mechanism of co-expression of diketide-CoA synthase and multiple curcumin synthases in turmeric rhizome has a significant effect on curcuminoid balance in different turmeric cultivars. Further experiment will explore more insights; however, association-based test results from this experiment can be useful in improving curcuminoid yield in turmeric rhizome or in vitro through the application of genetic engineering and biotechnology.

**Graphical abstract:**

Associating gene expressions with curcuminoid biosynthesis in turmeric 
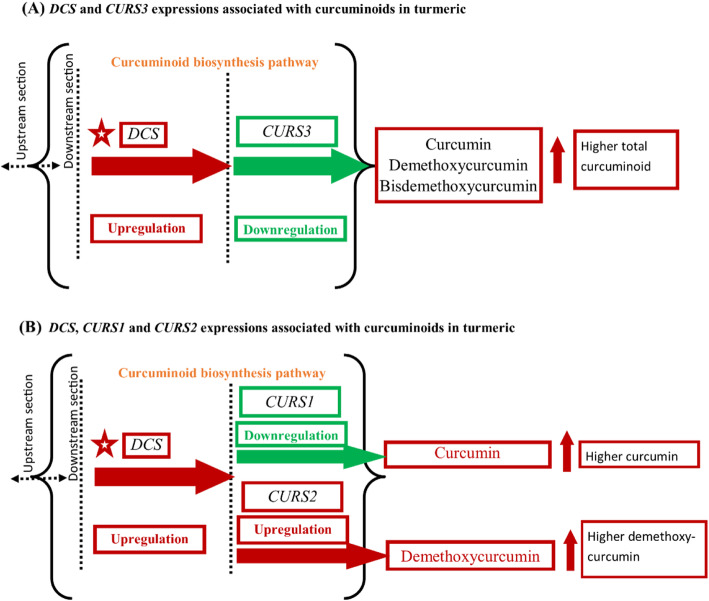

## Background

Turmeric (*Curcuma longa* L. syn. *Curcuma domestica* Val.) is a rhizomatous, herbaceous, and perennial plant belonging to the Zingiberaceae family which includes almost 110 species [1]. Turmeric is also known by different names in different countries such as *Haldi* in India and *Besar* in Nepal. Turmeric is widely cultivated in the tropical and subtropical regions of the world including India, China, Bangladesh, Nepal, Sri Lanka, Pakistan, Asian subcontinents, and the Caribbean and Latin American regions [2]. The use of turmeric rhizome is mentioned in the Vedic culture in Indian subcontinents and still has extensive use as a culinary spice, coloring agent, food preservative, natural dye in the food industry, cosmetics, medicine, and religious rituals [3, 4]. The first evidence of biological effects of turmeric have been attributed to its constituent curcumin, discovered and isolated almost two centuries ago [5]; however, recent studies suggest that most of the pharmaceutical properties of turmeric depend on its biologically active constituents, curcuminoids viz curcumin, demethoxycurcumin, and bisdemethoxycurcumin [6]. The turmeric rhizome contains 3–5% curcuminoids and up to 5% essential oils and resins [7] where commercially available turmeric powder is generally composed of about 77% curcumin, 17% demethoxycurcumin, and 3% bisdemethoxycurcumin [6]. Curcumin, a major curcuminoid from turmeric rhizome, is proven for its significance as anti-inflammatory, antioxidant, antimutagenic, antidiabetic, antibacterial, antiviral, antifungal, hepatoprotective, kidney disease treatment, expectorant, anti-cancerous, treating conditions like arthritis, inflammation to Alzheimer’s disease, and influencing gene transcription and expression, cell signaling pathway, and epigenetic mechanisms [3, 8–12]. Turmeric is a minor crop in terms of its contribution towards food security; however, the curcuminoids from this spice crop are attractive targets for metabolic engineering and pharmaceutical applications [13].

Curcuminoids are biologically synthesized through a phenylpropanoid pathway in turmeric [14]; however, several studies were also carried out successfully for producing curcuminoids in vitro through an artificial curcuminoid biosynthesis pathway [13, 15, 16]. There are several other upstream section enzyme genes involved in the curcuminoid biosynthesis pathway which catalyze the synthesis of early substrates (feruloyl-CoA/*p*-coumaroyl-CoA) [14, 17, 18]; however, downstream section enzyme genes involved in the curcuminoid biosynthesis pathway play a major role in catalyzing the formation of curcuminoid scaffold in turmeric rhizome. Diketide-CoA synthase (*DCS*), a type III PKS, and multiple curcumin synthases (*CURS*s) viz curcumin synthase1 (*CURS1*), curcumin synthase2 (*CURS2*), and curcumin synthase3 (*CURS3*) involved in the downstream section of the curcuminoid biosynthesis pathway in turmeric were identified and characterized where *DCS* synthesizes feruloyldiketide-CoA/*p*-coumaroyldiketide CoA and *CURS1*, *CURS2*, and *CURS3* then converts diketide-CoA esters into different curcuminoid scaffolds viz curcumin, demethoxycurcumin, and bisdemethoxycurcumin [19, 20]. The authors also reported that relative expression of *DCS*, *CURS1*, and *CURS2* were higher in rhizome than leaf whereas *CURS3* expression was almost similar in rhizome and leaf. The gene expression studies suggested that the content of curcuminoids in turmeric cultivars depends on the expression balance of these four major enzyme genes (*DCS*, *CURS1*, *CURS2*, and *CURS3*) in turmeric rhizome in addition to the availability of the substrates for curcuminoid scaffold biosynthesis. Transcriptomics studies in turmeric have shed light on the identification and validation of multiple curcumin synthases involved in the curcuminoid biosynthetic pathway in turmeric [17, 18, 21]. More novel type III PKS genes have been identified recently, such as *CLPKS9* and *CLPKS10* [22], *CLPKS1*and *CLPKS2* [18], and *CLPKS11* [23] which may have a potential role in curcuminoid scaffold biosynthesis; however, the difference in the contents of curcuminoids among the species can be explained by the changes in the expression of genes encoding diketide-CoA synthase and multiple curcumin synthases at the branching point of the curcuminoid biosynthesis pathway in turmeric [24]. The variation in curcuminoid content among the various lines and cultivars of *C. longa* was reported to be caused by hybridization and introgression [25], agro-climatic variation [26], genotype and environment interaction [27], and microenvironment (below soil surface near root zone) and macroenvironment (above soil surface) interaction [28]; however, the effects of tissue-specific gene expressions on curcuminoid biosynthesis are still unknown. Gene expressions of diketide-CoA synthases and multiple curcumin synthases in turmeric were also found to be influenced by tissue-specific (leaves and rhizome) and temporal (growth stages) variations within a cultivar [29]; this may have an influence on curcuminoid balance. *CURS* expression was found regardless of the variation in the curcumin content in different turmeric cultivars at different agroclimatic regions, and expressions were positively correlated with curcumin content in turmeric cultivars within an environment at different stages of growth [30]; however, the effect of individual *DCS* and *CURS* gene expression on curcuminoid yield is still unexplored. The first-stage study of this experiment revealed that differential *DCS* and *CURS* expressions can be predictively associated with curcuminoid content in different turmeric cultivars to study the relationship between target gene expressions and curcuminoid yield in turmeric rhizome [31]. Specific genes associated with target quantitative traits are commonly known as trait-associated genes (TAGs) which can be defined by the robust linear regression analysis through an association test between continuous traits and mRNA expression [32]. Huber’s M-estimator-based robust regression method [33] is useful for identifying TAGs. In this experiment, we studied *DCS* and *CURS* gene expressions in association with variable curcuminoid content in turmeric rhizome at different stages of growth to find out the impact of individual gene expression on curcuminoid yield in terms of magnitude and direction using a statistical model. Gene expressions or transcript abundance was regarded as genotypic values whereas associated changes in curcuminoids were regarded as phenotypic values at different growth stages while performing the association-based test. The association-based test results from this experiment can also be useful for improving curcuminoid yield in turmeric rhizome or in vitro through the application of genetic engineering and biotechnology.

## Methods

### General experimental conditions

Rhizome samples of turmeric cultivars (GNT-2, Pratibha, and NDH-98) were selected randomly for gene expression study and curcuminoid analysis at three different growth stages in field conditions. The first stage was determined to be the active vegetative growth stage (SI, 4 months after planting), the second stage was at the active rhizome development stage (SII, 5 months after planting), and the third stage was at the maturity or senescence stage (SIII, 6 months after planting). Three biological samples (rhizomes) from each cultivar at three stages of growth under study were taken out of the soil for RNA isolation, isolated RNA was reverse transcribed into complementary deoxyribonucleic acid (cDNA), and gene expression was studied through RT-qPCR in the Department of Genetics and Plant Breeding and Biotechnology Laboratory at NAU, Gujarat, India, during 2017–2018. The cultivars used in this experiment were denoted as biological sets NDH-98 (N), GNT-2 (G), and Pratibha (P). From each biological sample, two technical replicates were utilized for further downstream analysis through qPCR. The gene expression results obtained from the RT-qPCR assay were further used for association tests with curcuminoid yield in turmeric rhizome. Plant sampling procedures for RNA isolation were carried out following the recommended guidelines [34]. Technical sampling procedures and RT-qPCR procedures were followed as per the MIQE guidelines for the gene expression study [35, 36].

### Rhizome sampling and curcuminoid content analysis

At each stage of study viz SI, SII, and SIII, rhizome fingers were excised from the mother rhizome with a sterile knife and washed, packed into aluminum foil along with identification number, and immediately dipped into liquid nitrogen (− 196 °C) container for RNA isolation, cDNA synthesis, and gene expression study in the laboratory. The remaining portions of individually sampled rhizomes were cleaned, sliced into pieces, sun-dried for 5–7 days, and made into a fine powder using the grinder for curcuminoid content analysis (curcumin, demethoxycurcumin, and bisdemethoxycurcumin) through HPLC using individual curcuminoid standards (Sigma Aldrich) at the Food Quality Testing Laboratory (FQTL), NAU, Navsari, Gujarat. For curcuminoid analysis, 1.0 g turmeric powder of each cultivar was dissolved in 50 ml of 100% methanol separately and then mixed and vortexed for 30 min. The solution is then filtered and injected in the Thermo HPLC Surveyor instrument (Thermo Scientific) for individual curcuminoid measurement at 425-nm wavelength. Laboratory analysis was performed at room temperature (25–30 °C).

### RNA isolation and cDNA synthesis

Total RNA was isolated at three different growth stages (SI, SII, and SIII) of three turmeric cultivars under study using the RNeasy® Plant Mini Kit (Qiagen, Germany) involving on-column genomic DNA digestion step of DNase I (Qiagen) treatment following the manufacturer’s protocol. The integrity and size distribution of the total RNA purified were checked by 1.2% denaturing formaldehyde agarose (FA) gel electrophoresis and staining with ethidium bromide visualizing two sharp bands of 28S rRNA to 18S RNA in a 2:1 ratio. RNA purity check and quantification were done using the Nanodrop 2000 instrument (Thermo Scientific) which showed the acceptable purity for each biological sample (A_260_/A_280_ of 1.9 to 2.1). From the total RNA, the mRNA with the poly-A tail was reverse transcribed into cDNA using oligo dT primers during the reverse transcription step. cDNA was synthesized in 20 μl reaction using 2 μl of total isolated RNA (1 ng in final solution) with 4 μl of 5X cDNA synthesis buffer (1X final), 2 μl of 2X dNTP mix (500 μM each), 1 μl of anchored oligo dT primer (for mRNA reverse transcription), 1 μl RT enhancer, 1 μl verso enzyme mix, and water up to 20 μl. cDNA synthesis protocol involved a single 30-min cycle of polymerase enzyme activity at 42 °C followed by one additional cycle of 2 min at 95 °C for inactivation of enzyme (Verso cDNA Synthesis Kit, Thermo Scientific).

### Quantification of transcript abundance through RT-qPCR assays

RT-qPCR assays at three different stages were carried out along with gene-specific and reference gene primers (Additional file 1: Table S2) which were validated in turmeric RNA samples. All the qPCR assays of four target genes as well as reference genes at each stage were included in a single 96-well plate (Thermo Scientific) for minimizing error while quantifying the abundance of cDNA in technical replicates of each biological sample. A standard qPCR protocol and plate setup were performed on CFX96 real-time PCR thermocycler using the CFX Manager software (Bio-Rad). For finding out transcript abundance in different turmeric samples, qPCR assay comprised 10 μl of 2X PowerUp™ SYBR™ Green Master Mix (Applied Biosystems), 0.5 μM of each gene-specific forward and reverse primers along with reference gene *Actin*, 2 μl of 10-fold diluted cDNA as a template, and remaining quantity of nuclease-free water (HiMedia) to make a final volume of 20 μl. qPCR conditions for gene expression study were as follows: heat-labile uracil-DNA glycosylase (UDG) activation at 50 °C for 2 min followed by Dual-Lock™ DNA polymerase activation at 95 °C for 2 min, and 40 cycles of denaturation at 95 °C for 15 s followed by combined anneal/extend temperature of 60 °C for 1 min. For studying the specificity of the products, the final melting curve analysis of 65 to 95 °C with a 0.5 °C increment was used according to the manufacturer’s protocol (Bio-Rad, CFX96 instrument), and the products were also confirmed on 1.2% agarose gel electrophoresis. Quantification cycle (*C*_q_) and normalized gene expression values obtained in the CFX Manager software were used for further analysis. Negative controls (no template control (NTC)) were also included to detect any false positives during reverse transcription (RT) and qPCR assays. The data generated along with the *C*_q_ values were subjected to the CFX Manager (v.3.1, Bio-Rad) software package which includes mathematical models for estimating the relative quantification and normalization of qPCR data for gene expression analysis [37, 38]. Gene study feature in the CFX Manager software was set to a regulation threshold of 2-fold change and a *p* value threshold of 0.05 for finding out any significant variations in the gene expressions. *DCS* and *CURSs* melt peak curves were studied for the specificity of the products, and gel electrophoresis was also performed for the validation of the qPCR products using the reference gene *Actin*.

### Association-based test between gene expressions and curcuminoids

In this experiment, diketide-CoA synthase (*DCS*) and multiple curcumin synthases (*CURS1*, *CURS2*, and *CURS3*) or *CURS*s gene expression patterns were studied at three different growth stages viz SI (active vegetative stage), SII (active rhizome development stage), and SIII (maturity or senescence stage) among three turmeric cultivars significantly differing in curcuminoid (curcumin, demethoxycurcumin, and bisdemethoxycurcumin) contents in the rhizome. The robust regression analysis was used by taking normalized gene expression values from the RT-qPCR analysis as explanatory or independent variables and curcuminoid contents (curcumin, demethoxycurcumin, bisdemethoxycurcumin, and total curcuminoid) obtained from HPLC analysis as a response or dependent variables for identifying genes that are more closely associated with curcuminoid biosynthesis in turmeric, also known as trait-associated genes (TAGs). A separate association-based test was also done for the total curcuminoid content in turmeric rhizome which was estimated by summing up the individual curcuminoid content in each turmeric rhizome at each growth stage. The association between curcuminoids and mRNA expressions was analyzed using Huber’s M-estimator-based ROBUSTREG procedure [39] in SAS® University Edition (SAS® Studio 3.71, SAS® Institute Inc.). The general linear regression model for the association-based test [32] using gene expression data was as follows:

Trait_*i*_ = *β*_0_ + *β*_1_ Expression_1*i*_ + … + *β*_*p*_ Covariate_*pi*_ + *ε*_*i*_, *ε*_*i*_~(*N*, *σ*^2^) 

where *i* represents the individuals; Expression_*i*_ indicates the normalized gene expression value; Trait_*i*_ represents each continuous trait such as curcumin, demethoxycurcumin, bisdemethoxycurcumin, and total curcuminoid; Covariate_*pi*_ represents the covariate effects of factors other than error factors (*ε*_*i*_); beta (*β*) in a linear regression model is a standardized coefficient indicating the magnitude of the correlation between an independent and dependent variable; *N* and *σ*^2^ represent the analysis of variance assumption of normal distribution and common standard deviation, respectively. Additional tests like the rho (*ρ*) test based on the robust *F*-test and *R*_*n*_^2 test based on the Wald-type tests were also carried out to measure the association of covariates on response variables. The SAS codes and dataset used during the data analysis are given in Additional file 1: Annex 1.

## Results

### Curcuminoid analysis in turmeric cultivars

Based on the high-performance liquid chromatography (HPLC) analysis, turmeric cultivars “GNT-2” and “Pratibha” were not significantly different for curcumin and total curcuminoid content whereas “NDH-98” was with relatively lower curcumin and lower total curcuminoid content than “GNT-2” and “Pratibha” (Table 1). There was no significant difference among the cultivars for demethoxycurcumin content; however, the highest bisdemethoxycurcumin was in “GNT-2” followed by “Pratibha” and below detection level in “NDH-98” turmeric rhizome. Irrespective of the curcuminoid contents, the rhizome yield of short-growth-duration cultivar “NDH-98” was the highest (33–34 t ha^−1^) followed by long-growth-duration cultivars “GNT-2” (28–29 t ha^−1^) and “Pratibha” (23–24 t ha^−1^) (Table 1). The results showed that the higher turmeric rhizome yield does not necessarily mean higher curcuminoids in the turmeric cultivars. The chromatograms showing individual curcuminoid detection in the HPLC system are given in Additional file 2.

**Table 1:** Curcuminoid contents in turmeric cultivars at different stages under study

Sample ID	Curcumin (%)	Demethoxy curcumin (%)	Bisdemethoxycurcumin (%)	Total curcuminoid (%)	Rhizome yield (t ha^−1^)	Growth duration (days)
NDH-98_SI	0.27	0.76	BDL	1.03		
NDH-98_SII	0.1	0.36	BDL	0.46		
NDH-98_SIII	0.04	0.08	BDL	0.12		
Average NDH-98	0.14b (25%)	0.40a (75%)	BDL	0.54b (100%)	33–34	240
GNT-2_SI	2.31	0.71	1.14	4.16		
GNT-2_SII	0.76	0.07	0.15	0.98		
GNT-2_SIII	0.81	0.18	0.24	1.23		
Average GNT-2	1.29a (61%)	0.32a (15%)	0.51a (24%)	2.12a (100%)	28–29	256
Pratibha_SI	0.56	0.11	0.14	0.81		
Pratibha_SII	1.29	0.17	0.14	1.6		
Pratibha_SIII	1.33	0.22	0.24	1.79		
Average Pratibha	1.06a (76%)	0.17a (12%)	0.17b (12%)	1.40ab (100%)	23–24	256

“a” and “b” are the significance letters which indicate that values with the same letters in a column are statistically not significant from each other at *p* < 0.05. [NDH-98 has lower curcumin (25%), higher demethoxycurcumin (75%), and negligible bisdemethoxycurcumin; GNT-2 has higher curcumin (61%) and lower bisdemethoxycurcumin (24%) and demethoxycurcumin (15%); Pratibha has higher curcumin (76%) and lower demethoxycurcumin (12%) and bisdemethoxycurcumin (12%); GNT-2 and Pratibha have similar and higher total curcuminoid yield than NDH-98]

NDH-98, GNT-2, and Pratibha turmeric cultivars

*BDL* Below the detection limit during HPLC analysis, *SI* 4 months after planting, *SII* 5 months after planting, *SIII* 6 months after planting

### Gene expressions in turmeric rhizome at different growth stages

**Fig. 1:**
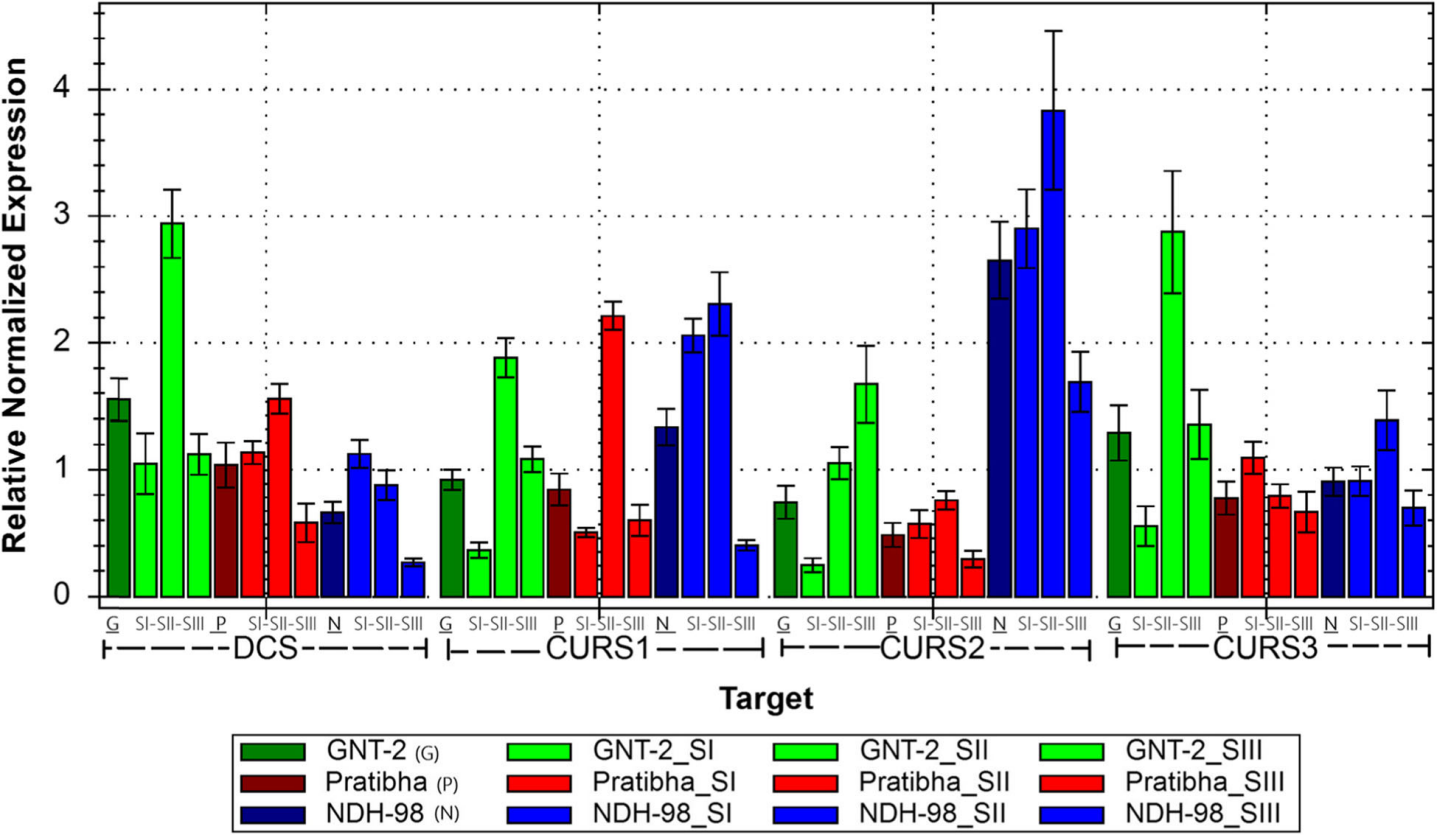
Comparative gene expression analysis in turmeric cultivars at three stages of growth. Gene expressions were normalized to the reference gene *Actin*. Green colored bars represent GNT-2, red-colored bars represent Pratibha, and dark blue colored bars represent NDH-98. The first bar in each gene family is the average of the three stages SI, SII, and SIII of each cultivar, i.e., GNT-2 (G), Pratibha (P), and NDH-98 (N). In NDH-98, *DCS* expression is decreasing at all stages of growth from SI to SIII, *CURS1*, and *CURS2* expression is higher at SI and SII and decreased at SIII whereas higher expression of *CURS3* at SII stages under study. In the case of GNT-2, *DCS*, *CURS1*, and *CURS3* are highly expressed at SII than SI and SIII, the *CURS2* expression level is increasing throughout the growth stages from SI to SIII where *DCS* and *CURS3* have almost equal expression level at SII in GNT-2. For Pratibha, *DCS*, *CURS1*, and *CURS2* gene expressions are higher at SII as compared with SI and SIII, and *CURS3* gene expression is decreasing at all stages of growth from SI to SIII. A significant amount of gene expression variation was observed during SII (5 months after planting) among the cultivars

There were no off products in the qPCR analysis based on the melt peak curves which was further confirmed by gel electrophoresis showing a single band of PCR product, and the data was considered reliable for further analysis. The results of the qPCR analysis are given in Additional file 2. Comparing the gene expression profiles in different turmeric cultivars, *DCS* (2.94-fold) and *CURS3* (2.87-fold) expressions were upregulated at SII as compared with SI and SIII; however, *CURS1* (1.88-fold) and *CURS2* (1.05 fold) were expressed at a lower level in GNT-2. In NDH-98, *DCS* (0.88-fold) and *CURS3* (1.39-fold) expressions were lower; however, *CURS1* (2.30-fold) and *CURS2* (3.83-fold) expressions were higher at SII as compared with SI and SIII. In Pratibha, *DCS* (1.56-fold), *CURS2* (0.76-fold), and *CURS3* (0.79-fold) were expressed at a lower level; however, *CURS1* (2.22 fold) expression was upregulated at SII as compared with SI and SIII (Fig. 1 and Additional file 1: Table S1). Generally, the gene expressions were increasing from active vegetative stage to rhizome development stage (SI to SII) and decreasing at maturity or senescence stage (SIII) under study except that *DCS* expression was decreasing throughout the growth stages in NDH-98, *CURS2* expression was increasing throughout the growth stages in GNT-2, and *CURS3* expression was decreasing throughout the growth stages in Pratibha (Fig. 1). These patterns in each cultivar might have some significant role to play for curcuminoid yield in different turmeric cultivars.

### Pooled gene expressions in turmeric cultivars

**Fig. 2:**
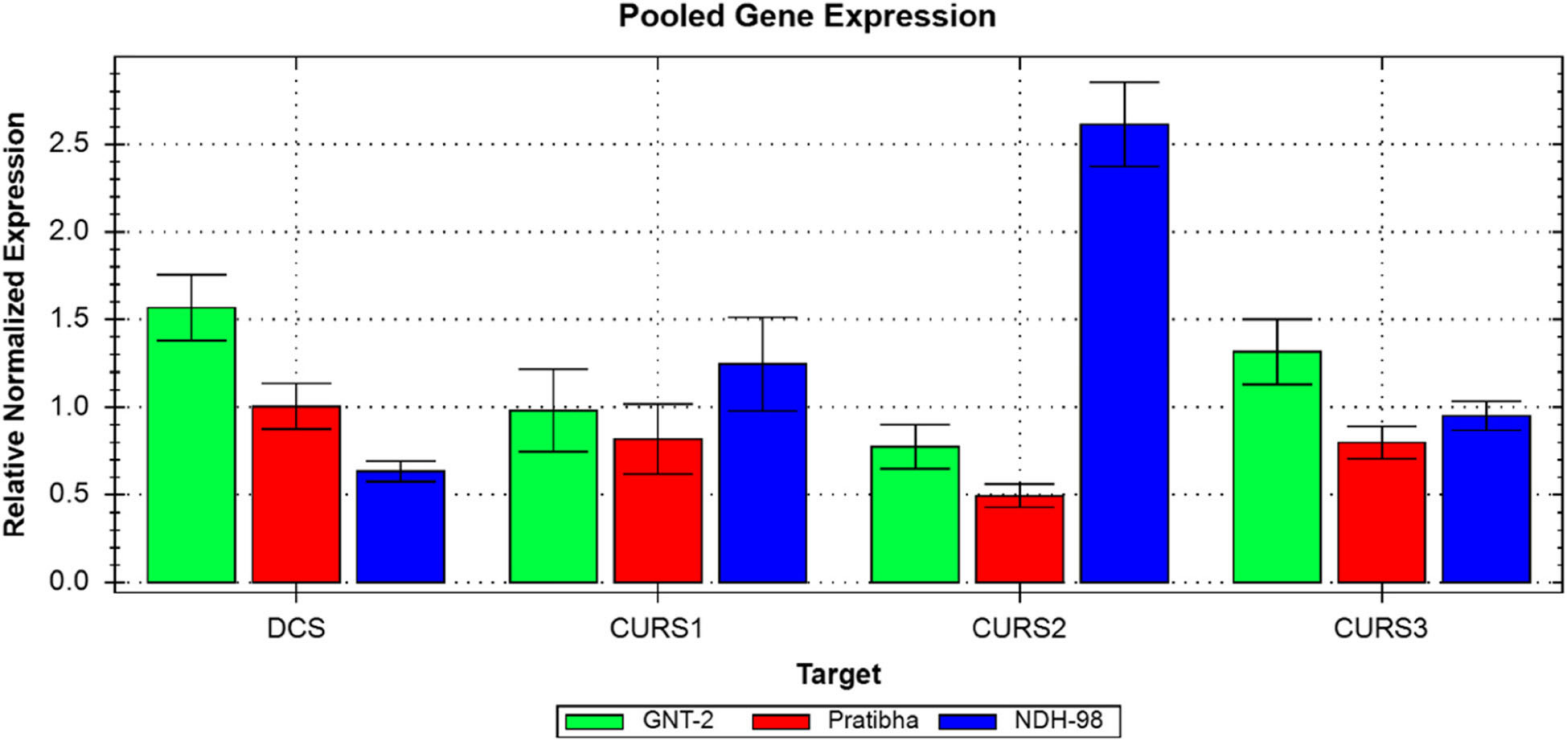
Pooled gene expression in three turmeric cultivars. GNT-2, Pratibha, and NDH-98 are turmeric cultivars. *DCS*, diketide-CoA synthase. *CURS1*, *CURS2*, and *CURS3* are multiple curcumin synthases. Pooled gene expressions are the averages of three stage (SI, SII, and SIII) expressions in each cultivar. *DCS* and *CURS3* expressions are higher in GNT-2, a higher curcuminoid yielding cultivar, whereas *CURS1* and *CURS2* expressions are higher in NDH-98, a lower curcuminoid yielding cultivar

The highest *DCS* expression was in higher curcumin yielding cultivar GNT-2 (1.55-fold) followed by Pratibha (1.04-fold) and the least in low curcumin yielding cultivar NDH-98 (0.66-fold) over the growth stages under study. *CURS1* and *CURS2* gene expressions were highest in NDH-98 (1.34-fold and 2.65-fold, respectively) followed by GNT-2 (0.92-fold and 0.74-fold, respectively) and Pratibha (0.84-fold and 0.49-fold, respectively). *CURS3* expressions were lower than *DCS* expressions in all the cultivars over growth stages with the highest expression in GNT-2 (1.29-fold) followed by NDH-98 (0.91-fold) and Pratibha (0.77-fold). Among all the genes under study, *CURS2* expressions were lower in Pratibha (0.48-fold) and GNT-2 (0.74-fold), however significantly higher in NDH-98 (2.65-fold). The results of pooled gene expressions over growth stages are in Fig. 2 and Additional file 1: Table S1. The results showed that GNT-2 and Pratibha, higher curcuminoid yielding cultivars, had a higher *DCS* expression with relatively lower *CURS1*, *CURS2*, and *CURS3* expressions opposite to that of NDH-98, relatively lower curcuminoid yielding cultivar, which depicted lower *DCS* expression with relatively higher *CURS1*, *CURS2*, and *CURS3* expressions (Fig. 2). There was no significant change in *DCS*, *CURS1*, *CURS2*, and *CURS3* gene expressions between GNT-2 and Pratibha (higher curcumin and total curcuminoid yielding cultivars) at all stages of growth: however, their expressions differed in NDH-98 (lower curcumin and total curcuminoid yielding cultivar) (Table 2). *DCS* expression was found downregulated (− 2.35- and –1.57-fold) whereas *CURS2* expression was found upregulated (3.57- and 5.46-fold) in lower curcumin and lower total curcuminoid yielding cultivar (NDH-98) as compared with higher curcumin and higher total curcuminoid yielding cultivars (GNT-2 and Pratibha) and vice versa (Table 2). There was no significant change in the *CURS3* expression (< 2-fold change) among the cultivars.

**Table 2:** *DCS* and *CURS*s gene regulation in turmeric rhizome over growth stages

Target	Experimental sample	Regulation	*p* value	Exceeds *p* value threshold	Compared to regulation threshold	Control sample
*Actin*	GNT-2	NA	0.790985	Yes	No change	Pratibha
*CURS1*	GNT-2	1.09	0.179227	Yes	No change	Pratibha
*CURS2*	GNT-2	1.53	0.192329	Yes	No change	Pratibha
*CURS3*	GNT-2	1.66	0.003796	No	No change	Pratibha
*DCS*	GNT-2	1.50	0.369600	Yes	No change	Pratibha
*Actin*	NDH-98	NA	0.496892	Yes	No change	Pratibha
*CURS1*	NDH-98	1.59	0.730277	Yes	No change	Pratibha
*CURS2*	NDH-98	5.46	0.000001	No	Upregulated	Pratibha
*CURS3*	NDH-98	1.17	0.395052	Yes	No change	Pratibha
*DCS*	NDH-98	− 1.57	0.044860	No	No change	Pratibha
*Actin*	NDH-98	NA	0.689920	Yes	No change	GNT-2
*CURS1*	NDH-98	1.45	0.036328	No	No change	GNT-2
*CURS2*	NDH-98	3.57	0.000027	No	Upregulated	GNT-2
*CURS3*	NDH-98	− 1.43	0.022002	No	No change	GNT-2
*DCS*	NDH-98	− 2.35	0.000368	No	Downregulated	GNT-2

“+” or no sign in the regulation column represents upregulation (higher expression) of a gene in the current sample as compared with the control sample

“−” sign indicates downregulation (lower expression) of a gene in the current sample as compared with the control sample

Upregulation of a “gene” in experimental sample as compared with control also indicates the downregulation of that same “gene” in the control sample as compared with experimental sample and vice versa. Gene expressions at different stages of growth viz SI, SII, and SIII for each cultivar were pooled together for comparative gene regulation study

### Cluster analysis of *DCS* and *CURSs* gene expressions

**Fig. 3:**
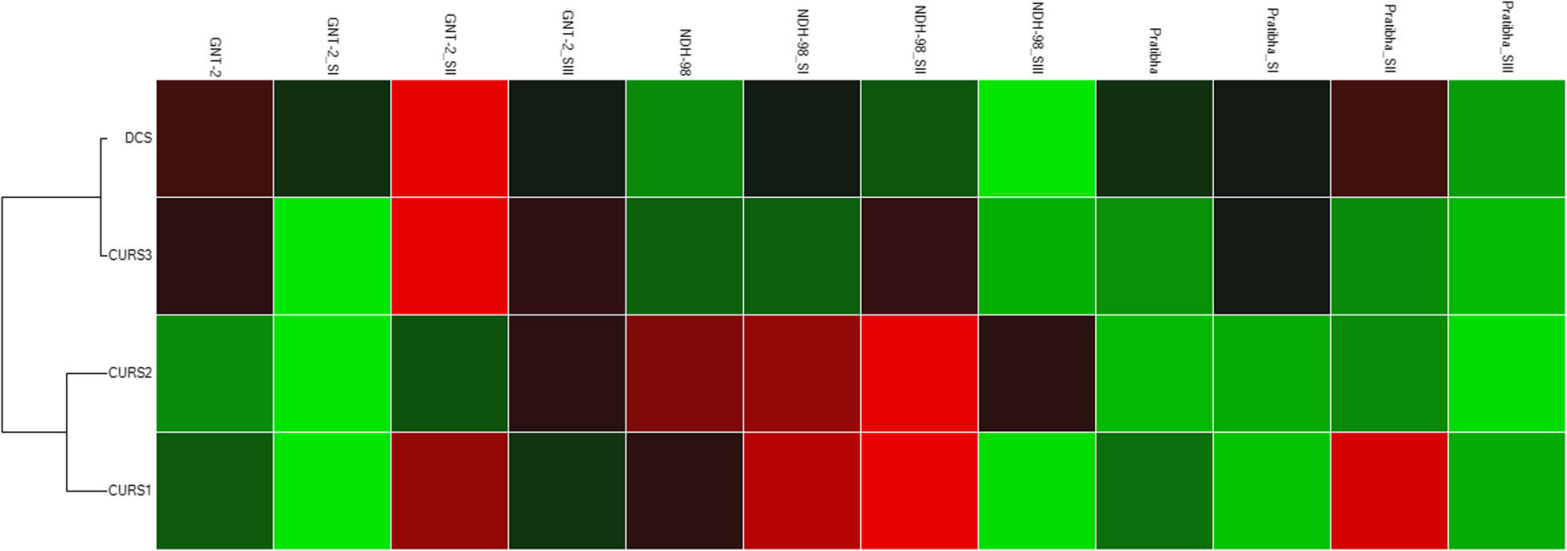
Cluster gram showing the association between the gene expressions in turmeric at three growth stages. NDH-98, GNT-2, and Pratibha are three turmeric cultivars. SI, 4 months after planting; SII, 5 months after planting; SIII, 6 months after planting. Upregulation (higher expression) is shown by a red square, downregulation (lower expression) by a green square, and no regulation by a black square. The lighter the shade of color, the greater the relative expression difference. Gene expression patterns of *DCS* and *CURS3* were more closely associated and distant from *CURS1* and *CURS2* gene expression patterns among the genotypes at all stages of growth. The upregulation of most of the genes was found at SII as compared with other states under study. GNT-2, NDH-98, and Pratibha expressions are the average expressions of all three stages

Cluster analysis of the gene expressions of three turmeric cultivars at three different growth stages revealed that *DCS* and *CURS3* expression patterns were similar but distinct from *CURS1* and *CURS2* expression patterns in turmeric rhizome (Fig. 3). Similar results were obtained when gene expression was studied in two turmeric cultivars, GNT-2 and NDH-98, after 6 months of planting in field conditions [31].

### *DCS* and *CURSs* gene expressions associated with curcuminoid biosynthesis in turmeric

There was a significant effect of *DCS*, *CURS1*, and *CURS3* gene expressions on curcumin content at *p* < 0.01. *DCS* had a positive linear association whereas *CURS1* and *CURS3* expressions had a negative linear association with curcumin. The covariate effect of *CURS1* and *CURS2* expressions on curcumin was also found significant at *p* < 0.01 although the correlation was significant at *p* < 0.08. Thus, *DCS* had a significantly positive whereas *CURS1* and *CURS3* expressions had a significantly negative association with curcumin yield in turmeric rhizome. Co-regulated expressions of *CURS1* and *CURS2* were also found significantly associated with curcumin synthesis in turmeric rhizome (Table 3).

**Table 3:** Association-based test of *DCS* and multiple curcumin synthases with curcumin

Parameter	Class label	Main effects			*CURS1* and *CURS2* as a covariate		
		Estimate	Chi-square	Pr > ChiSq	Estimate	Chi-square	Pr > ChiSq
Intercept		1.1091	486.32	< .0001	1.4185	327.39	< .0001
Growth stage	SI	0.1039	4.05	0.0442	− 0.1792	4.53	0.0333
Growth stage	SII	0.4701	57.26	< .0001	0.0073	0.01	0.936
Growth stage	SIII	0			0		
Genotype	GNT-2	1.0375	342.2	< .0001	1.1168	161.13	< .0001
Genotype	NDH-98	− 0.2774	14.84	0.0001	− 0.616	61.29	< .0001
Genotype	Pratibha	0			0		
*DCS*		0.9126	82.3	< .0001	0.6182	26.03	< .0001
*CURS1*		− 0.3392	12.48	0.0004	0		
*CURS2*		− 0.0033	0.01	0.9371	0		
*CURS3*		− 1.3922	335.16	< .0001	− 1.2216	153.92	< .0001
Reduced parameter robust linear test for curcumin							
Rho					4.1143	5.16	0.0759
*R*_*n*_^2					27.0998	27.1	< .0001

Pr < ChiSq approximately predicts the associated probability level after the selected variable has been entered or removed

*Pr* Probability, *ChiSq* Chi-square, *Rho* association or correlation, *R*_*n*_*^2* robust Wald-type association test

For demethoxycurcumin, *DCS* and *CURS2* expressions had a significant positive association whereas *CURS3* expressions had a significant negative association with demethoxycurcumin at *p* < 0.01. The covariate effect of *CURS1* and *CURS2* expressions on demethoxycurcumin was also found highly significant (*p* < 0.01). Thus, *DCS* and *CURS2* expressions had significant positive associations whereas *CURS3* expression had a significant negative association with demethoxycurcumin. Co-regulated expression of *CURS1* and *CURS2* also had a significant impact on the demethoxycurcumin content in the turmeric rhizome (Table 4).

**Table 4:** Association-based test of *DCS* and multiple curcumin synthases with demethoxycurcumin

Parameter	Class label	Main effects			*CURS1* and *CURS2* as a covariate		
		Estimate	Chi-square	Pr > ChiSq	Estimate	Chi-square	Pr > ChiSq
Intercept		− 0.1443	32.09	< .0001	− 0.1497	12.45	0.0004
Growth stage	SI	0.3705	200.58	< .0001	0.3895	73.06	< .0001
Growth stage	SII	0.2073	43.38	< .0001	0.2591	27.78	< .0001
Growth stage	SIII	0			0		
Genotype	GNT-2	0.4197	218.18	< .0001	0.4175	76.88	< .0001
Genotype	NDH-98	0.3327	83.19	< .0001	0.4616	117.48	< .0001
Genotype	Pratibha	0			0		
*DCS*		0.2596	25.95	< .0001	0.2913	19.72	< .0001
*CURS1*		0.0296	0.37	0.5431	0		
*CURS2*		0.0535	6.44	0.0111	0		
*CURS3*		− 0.4059	110.98	< .0001	− 0.4188	61.76	< .0001
Reduced parameter robust linear test for demethoxycurcumin							
Rho					10.1158	12.68	0.0018
*R*_*n*_^2					19.0271	19.03	< .0001

Pr < ChiSq approximately predicts the associated probability level after the selected variable has been entered or removed

*Pr* Probability, *ChiSq* Chi-square, *Rho* association or correlation, *R*_*n*_*^2* robust Wald-type association test

*DCS* (*p* < 0.09) depicted a positive effect whereas *CURS3* (*p* < 0.01) showed a significant negative effect on bisdemethoxycurcumin content. The covariate effects of *CURS1* and *CURS2* expression on bisdemethoxycurcumin were also found non-significant at *p* < 0.05. Thus, *DCS* expression had a positive effect and *CURS3* expression had a significant negative effect on bisdemethoxycurcumin content in turmeric rhizome (Table 5).

**Table 5:** Association-based test of *DCS* and multiple curcumin synthases with bisdemethoxycurcumin

Parameter	Class label	Main effects			*CURS1* and *CURS2* as a covariate		
		Estimate	Chi-square	Pr > ChiSq	Estimate	Chi-square	Pr > ChiSq
Intercept		0.3381	9.58	0.002	0.3586	12.19	0.0005
Growth stage	SI	0.1514	1.82	0.1771	0.1847	2.8	0.094
Growth stage	SII	0.1877	1.93	0.1644	0.0847	0.51	0.4768
Growth stage	SIII	0			0		
Genotype	GNT-2	0.5534	20.63	< .0001	0.6544	32.23	< .0001
Genotype	NDH-98	− 0.0154	0.01	0.9216	− 0.1401	1.85	0.1741
Genotype	Pratibha	0			0		
*DCS*		0.3727	2.91	0.0881	0.0342	0.05	0.8294
*CURS1*		− 0.2976	2.03	0.1537	0		
*CURS2*		0.0424	0.22	0.6389	0		
*CURS3*		− 0.618	13.99	0.0002	− 0.4367	11.46	0.0007
Reduced parameter robust linear test for bisdemethoxycurcumin							
Rho					1.5189	1.9	0.3859
*R*_*n*_^2					2.7031	2.7	0.2588

Pr < ChiSq approximately predicts the associated probability level after the selected variable has been entered or removed

*Pr* Probability, *ChiSq* Chi-square, *Rho* association or correlation, *R*_*n*_*^2* robust Wald-type association test

Only *DCS* and *CURS3* expressions had a significant effect (*p* < 0.01) on the total curcuminoid content in turmeric rhizome where *DCS* had a significant positive linear association and *CURS3* had a significant negative linear association with total curcuminoid. The covariate effect of *CURS1* and *CURS2* expression was found non-significant at *p* < 0.05. Thus, *DCS* had a significant positive and *CURS3* had a significant negative association with total curcuminoid yield in turmeric rhizome (Table 6).

**Table 6:** Association-based test of *DCS* and multiple curcumin synthases with total curcuminoid

Parameter	Class label	Main effects			*CURS1* and *CURS2* as a covariate		
		Estimate	Chi-square	Pr > ChiSq	Estimate	Chi-square	Pr > ChiSq
Intercept		1.0927	45.46	< .0001	1.2257	128.64	< .0001
Growth stage	SI	0.7981	23	< .0001	0.9247	63.49	< .0001
Growth stage	SII	1.0051	25.2	< .0001	0.8874	50.23	< .0001
Growth stage	SIII	0			0		
Genotype	GNT-2	2.1593	142.72	< .0001	2.4428	405.65	< .0001
Genotype	NDH-98	0.1852	0.64	0.4247	− 0.2492	5.28	0.0216
Genotype	Pratibha	0			0		
*DCS*		1.545	22.71	< .0001	0.5921	12.57	0.0004
*CURS1*		− 0.5897	3.63	0.0567	0		
*CURS2*		0.0863	0.41	0.5201	0		
*CURS3*		− 2.4214	97.63	< .0001	− 1.838	183.38	< .0001
Reduced parameter robust linear test for total curcuminoid							
Rho					3.1409	3.94	0.1396
*R*_*n*_^2					4.772	4.77	0.092

Pr < ChiSq approximately predicts the associated probability level after the selected variable has been entered or removed

*Pr* Probability, *ChiSq* Chi-square, *Rho* association or correlation, *R*_*n*_*^2* robust Wald-type association test

## Discussion

**Fig. 4:**
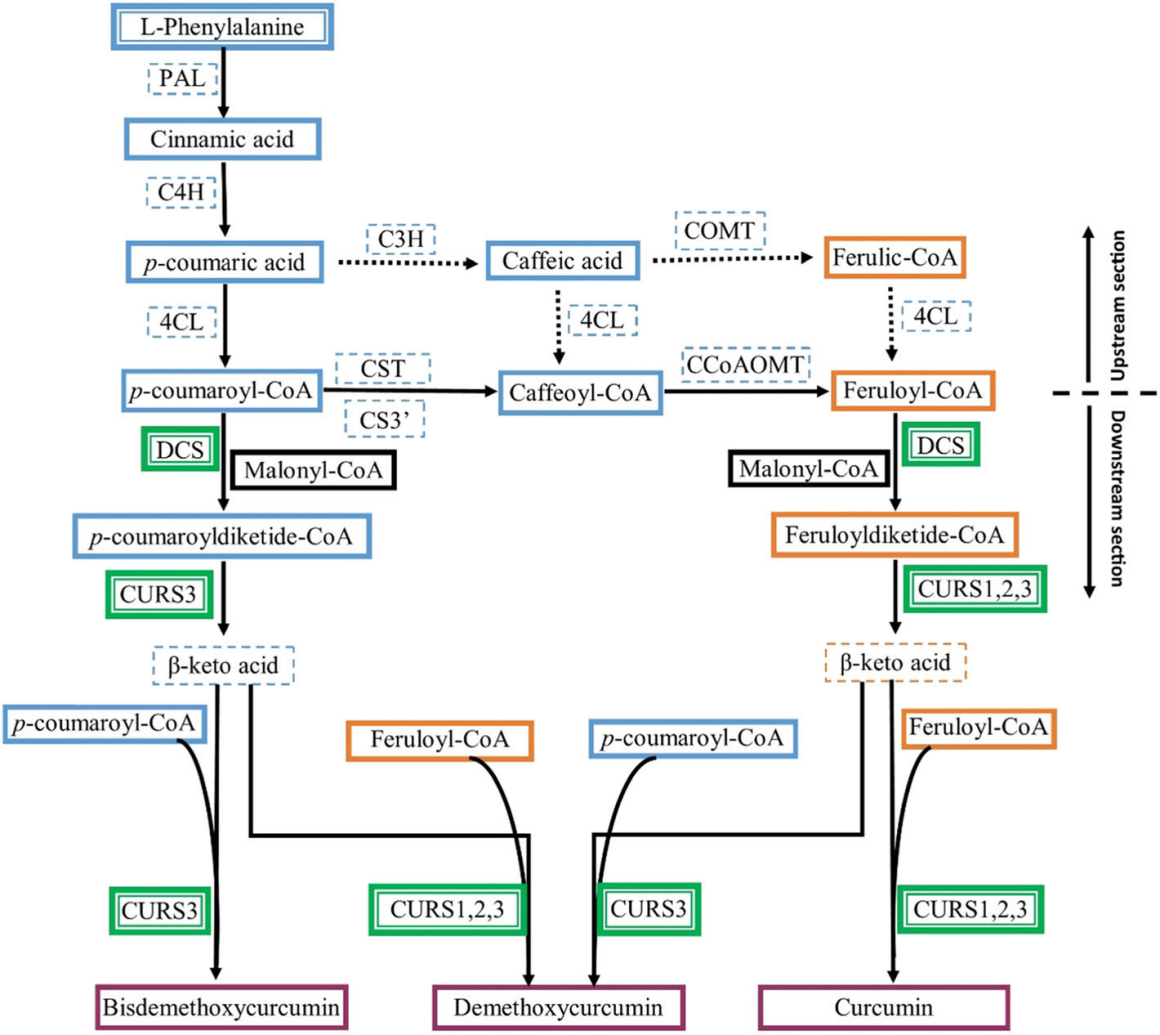
Curcuminoid biosynthesis pathway in turmeric [adapted and modified from Ramirez-Ahumada et al. [14] and Katsuyama et al. [19, 20]]. Cinnamic acid is synthesized from phenylalanine by phenylalanine ammonia lyase (*PAL*) and converted into coumaric acid by cinnamate-4-hydroxylase (*C4H*). Then, 4-coumarate-CoA ligase (*4CL*) converts coumaric acid into coumaroyl-CoA, and *p*-coumaroyl shikimate transferase (*CST*), *p*-coumaroyl 5-*O*-shikimate 3-hydroxylase (*CS3=H*), and caffeoyl-CoA-*O*-methyltransferase (*CCoAOMT*) convert it into feruloyl-CoA. Coumaroyl-CoA and feruloyl-CoA are then converted by diketide-CoA synthase (*DCS*) into diketide-CoAs by condensation with malonyl-CoA. In the end, multiple curcumin synthases (*CURS1*, *CURS2*, and *CURS3*) catalyze the formation of curcuminoids by condensing the diketide-CoAs with coumaroyl-CoA and feruloyl-CoA. Depending on the combination, different curcuminoids are produced, *namely* curcumin, demethoxycurcumin, and bisdemethoxycurcumin. The route indicated by solid arrows corresponds to the central phenylpropanoid pathway that occur in vivo, in *C. longa*

Gene expression studies can quantify the amount of functional mRNA (messenger ribonucleic acid) transcript in the experimental sample measured in terms of reverse-transcribed cDNA (complementary deoxyribonucleic acid) concentration which codes for amino acids and functional proteins. After the identification and characterization of multiple type III PKS enzyme genes involved in the curcuminoid biosynthesis pathway [19, 20], it is now possible to study the differential gene expression patterns using reverse transcription quantitative real-time polymerase chain reaction (RT-qPCR) assays in addition to protein-enzyme and product assays in different tissues of turmeric. The role of *DCS* and *CURS*s for curcuminoid biosynthesis in turmeric has been described by different studies (Fig. 4); however, the correlation or directional association between the gene expression level and curcuminoids level in turmeric rhizome is still unfolded. In this experiment, three cultivars differing in curcuminoid contents were evaluated for *DCS* and *CURSs* gene expressions, and normalized expression values are association tested with curcuminoid contents (curcumin, demethoxycurcumin, and bisdemethoxycurcumin) measured at three different growth stages in turmeric cultivars.

The curcuminoid analysis results showed that a higher turmeric rhizome yield does not necessarily mean higher curcuminoid contents in the turmeric cultivars. The curcuminoid contents were higher in long-growth-duration cultivar than short-growth-duration cultivar irrespective of the rhizome yield (Table 1). Differential gene expression patterns were also observed in three turmeric cultivars yielding different amounts of curcuminoids (curcumin, demethoxycurcumin, and bisdemethoxycurcumin). In general, higher gene expressions were during active vegetative and rhizome development stage (SI–SII) of turmeric cultivars than reduced expression at maturity or senescence stage (SIII) under study except that *DCS* expression was decreasing in NDH-98 and *CURS3* expression was decreasing in Pratibha throughout the growth stages (Fig. 1). Differential expression of *CURSs* genes was also reported to be positively correlated with the curcumin content in *Curcuma* spp. during the 3rd and 6th months (active vegetative and rhizome developmental stages); however, during the 9th month (maturity stage), the curcumin content increased and the putative *CURS*s gene expressions were downregulated [40]. These patterns in each cultivar might have some significant role to play for curcuminoid biosynthesis in different turmeric cultivars suggesting that the genotype and growth stages affect the *DCS* and *CURS*s expressions at different developmental stages in turmeric. It was also reported that the highest expression of multiple curcumin synthases in *Curcuma caesia* Roxb. was found at the peak of its vegetative stage and expression levels of *CURSs* decreased at the time of maturity [29]. Among the three cultivars under study, there was no significant difference in *DCS*, *CURS1*, *CURS2*, and *CURS3* gene expressions between GNT-2 and Pratibha (higher curcumin and total curcuminoid yielding cultivars); however, their expressions differed in NDH-98 (lower curcumin and total curcuminoid yielding cultivar) (Table 2). *DCS* expression was downregulated whereas *CURS2* expression was upregulated in lower curcumin and lower total curcuminoid yielding cultivar (NDH-98) as compared with higher curcumin and higher total curcuminoid yielding cultivars (GNT-2 and Pratibha) and vice versa (Table 2). There was no significant change in the *CURS3* expression (< 2-fold change) among the cultivars. *CURS3* expressions were also found lower in all the turmeric species differing in curcuminoid yield [24]. These results showed that there is a significant variation in *DCS* and *CURSs* gene expressions in turmeric rhizome also affected by genotype and growth stages.

The expressions of *CURSs* genes were also found to be affected by soil, climate, and harvesting time; however, *CURSs* expressions were found positively correlated with the curcumin content at a particular agroclimatic zone [30]. The expression of curcumin synthase genes (*CURS*s) and subsequent curcumin production varied at the different developmental stages of the plant in *Curcuma* spp. [29, 40]; however, we did not find any publications concerning *DCS* and *CURS*s expressions together in association with curcuminoid yield in turmeric to date. *CURS1* and *CURS2* expressions were also clustered as a distant group from *DCS* and *CURS3* in this experiment (Fig. 3). Additionally, the gene expression studies alone cannot explain the magnitude and direction of the association between genes (*DCS* and *CURS*s) and gene products (curcuminoids). Thus, a more robust association-based test (robust regression) was applied for finding out the magnitude and direction of the associations. *DCS* and *CURSs* expressions in turmeric rhizome samples at three different growth stages were studied in association with biologically synthesized curcuminoids viz curcumin, demethoxycurcumin, bisdemethoxycurcumin, and total curcuminoid contents in turmeric rhizome samples for identifying any significant association between them. A robust regression analysis using the M-estimator method was used for the association-based test which detects the outliers in the observed dataset and provides more precise information [32]. Association-based tests between the gene expression and curcuminoids revealed that differential expressions of *DCS*, *CURS1*, *CURS2*, and *CURS3* are responsible for the differential curcuminoid balance in turmeric rhizome, and their expressions are not always positively associated or correlated with curcuminoid yield. *DCS* expression had a significant positive but *CURS3* expression had a significant negative association with curcumin, demethoxycurcumin, bisdemethoxycurcumin, and total curcuminoid yield in turmeric rhizome (Table 7).

**Table 7:** Summary of the association-based tests between gene expression and curcuminoids

Gene ID	Association test results				Remarks
	Curcumin	Demethoxy-curcumin	Bisdemethoxy-curcumin	Total curcuminoid	
*DCS*	+, ***	+, ***	+, ¥	+, ***	*DCS* has a strong positive association with all the curcuminoids.
*CURS1*	−, ***	NS	NS	−, ¥	*CURS1* expression is negatively associated with curcumin.
*CURS2*	NS	+, *	NS	NS	*CURS2* expression has a positive association with demethoxycurcumin.
*CURS3*	−, ***	−, ***	−, ***	−, ***	*CURS3* expression has a strong negative association with all the curcuminoids.
*CURS1* and *CURS2*	¥	***	NS	¥	Covariate effect of *CURS1* and *CURS2* have significant impact on demethoxycurcumin.

*NS* non-significant

The results were adapted from Tables 3, 4, 5, and 6

+, positive linear association

−, negative linear association

¥, significant at *p* < 0.10

***p < 0.001; **p < 0.01; *p < 0.05

On the other hand, the covariate effects of *CURS1* and *CURS2* were also found to be associated with curcuminoid balance in turmeric rhizome. *CURS1* expression had a significant negative association with curcumin yield; however, *CURS2* expression showed a significant positive association with demethoxycurcumin. The co-regulated expression of *CURS1* and *CURS2* were also found significantly associated with demethoxycurcumin content in turmeric rhizome (Table 7). It was also reported that *DCS* and *CURSs* were capable of synthesizing curcuminoids through the major curcuminoid biosynthesis pathway in turmeric [19, 20] where *CURS3* is capable of synthesizing all three curcuminoids; however, *CURS1* and *CURS2* are mainly responsible for the curcumin and demethoxycurcumin biosynthesis [20]. The results in this study suggested that the level of expression of *DCS* and *CURSs* genes have different individual effects on curcuminoid biosynthesis.

The results showed that higher curcuminoid yield in turmeric rhizome is associated with higher *DCS* and relatively lower *CURS3* expressions, and vice versa. Similarly, higher *CURS1* expression is associated with lower curcumin yield; however, higher *CURS2* expression is associated with a higher demethoxycurcumin in turmeric rhizome, and vice versa (Table 7). This mechanism of co-expression of diketide-CoA synthase and multiple curcumin synthase genes involved in the curcuminoid biosynthesis pathway in turmeric rhizome revealed a significant effect on curcuminoid balance in different turmeric cultivars. Thus, diketide-CoA synthase and multiple curcumin synthase gene expressions are differentially associated with curcuminoid yield in turmeric rhizome, not always positively associated with individual curcuminoid. Further, enzymatic study regarding gene regulation of *DCS* and multiple curcumin synthases through genetic engineering and biotechnology approach will help to enhance curcuminoid content in turmeric rhizome or in vitro.

## Conclusions

Gene expressions of diketide-CoA synthase and multiple curcumin synthases involved in the curcuminoid biosynthesis pathway in turmeric rhizome are influenced by genotype and growth stages. *DCS* and *CURS3* expression patterns were closely associated and distinct from *CURS1* and *CURS2* expression patterns in turmeric rhizome. The association-based tests showed that *DCS* and *CURSs* gene expressions are not always positively associated with curcuminoid yield in turmeric rhizome. *DCS* expression had a significant positive but *CURS3* expression had a significant negative association with curcumin, demethoxycurcumin, bisdemethoxycurcumin, and total curcuminoid yield. Curcumin, a major curcuminoid, is also found negatively associated with *CURS1* expression. The co-regulated expressions of *CURS1* and *CURS2* were significantly associated with demethoxycurcumin content in turmeric rhizome. The gene regulation study of *DCS* co-expressed with multiple curcumin synthases (*CURS*s) and the overexpression of *DCS* through genetic engineering approach will help to enhance curcuminoid content in turmeric rhizome or in vitro for medicinal and industrial use.

## Supplementary Information

**Additional file 1: Table S1.** *DCS* and multiple curcumin synthases gene expressions at different stages in turmeric rhizome. **Table S2.** Gene specific and reference primers used in qPCR assay. **Table S3.** Duncan’s mean comparison of *DCS*, *CURS1*, *CURS2* and *CURS3* expressions among turmeric cultivars. **Annex 1.** SAS codes for association-based test using ROBUSTREG procedure.

**Additional file 2: Fig S1.** Chromatograms and calibration curves for curcumin, demothoxycurcumin and bisdemethoxycurcumin standards using HPLC. **Fig S2.** Chromatograms showing individual curcuminoid contents measured using HPLC at three stages of growth of three turmeric cultivars. **Fig S3.** Melt peak curves and gel electrophoresis results for *DCS*, *CURS1*, *CURS2*, *CURS3* and reference gene *Actin*. In melt peak curves, single peak of each gene specific product was observed in each sample. In gel image, N represents ‘NDH-98’; G represents, ‘GNT-2’; and P represents ‘Pratibha’ cultivars of turmeric under study.

## Data Availability

The dataset supporting the conclusions of this article are included within the article and its additional file: Additional file 1: Table S1, Table S2, and Annex 1.

## Abbreviations

CoA: Coenzyme A
cDNA: Complementary deoxyribonucleic acid
*CURS1*: Curcumin synthase 1
*CURS2*: Curcumin synthase 2
*CURS3*: Curcumin synthase 3
*DCS*: Diketide-CoA synthase
HPLC: High-performance liquid chromatography
mRNA: Messenger ribonucleic acid
PCR: Polymerase chain reaction
PKS: Polyketide synthase
RT-qPCR: Reverse transcription quantitative real-time PCR
SAS: Statistical Analysis System
UDG: Uracil DNA glycosylase
