# Supplementary material for: Associating gene expressions with curcuminoid biosynthesis in turmeric
Source: J Genet Eng Biotechnol. 2020 Dec 14;18:83. doi: 10.1186/s43141-020-00101-2 (PMC7736439; doi:10.1186/s43141-020-00101-2)
Supplement: Supplementary file 1 — Additional file 1: Table S1. DCS and multiple curcumin synthases gene expressions at different stages in turmeric rhizome. Table S2. Gene specific and reference primers used in qPCR assay. Table S3. Duncan’s mean comparison of DCS, CURS1, CURS2 and CURS3 expressions among turmeric cultivars. Annex 1. SAS codes for association-based test using ROBUSTREG procedure. [file 43141_2020_101_MOESM1_ESM.docx]

**Additional File 1:**

**Table S1. *DCS* and multiple curcumin synthases gene expressions at different stages in turmeric rhizome**

| Target | Sample | Expression | Expression SEM | Mean Cq | Cq SEM |
| --- | --- | --- | --- | --- | --- |
| *Actin* | GNT-2 | Reference | NA | 25.09 | 0.18736 |
| *Actin* | GNT-2_SI | Reference | NA | 24.52 | 0.29881 |
| *Actin* | GNT-2_SII | Reference | NA | 25.57 | 0.21444 |
| *Actin* | GNT-2_SIII | Reference | NA | 25.06 | 0.29479 |
| *Actin* | NDH-98 | Reference | NA | 25.93 | 0.11055 |
| *Actin* | NDH-98_SI | Reference | NA | 25.94 | 0.16374 |
| *Actin* | NDH-98_SII | Reference | NA | 25.78 | 0.21446 |
| *Actin* | NDH-98_SIII | Reference | NA | 26.05 | 0.22654 |
| *Actin* | Pratibha | Reference | NA | 25.00 | 0.20962 |
| *Actin* | Pratibha_SI | Reference | NA | 25.82 | 0.03141 |
| *Actin* | Pratibha_SII | Reference | NA | 24.17 | 0.15075 |
| *Actin* | Pratibha_SIII | Reference | NA | 25.19 | 0.33478 |
| *CURS1* | GNT-2 | 0.92030 | 0.08008 | 27.52 | 0.21660 |
| *CURS1* | GNT-2_SI | 0.36594 | 0.06076 | 28.29 | 0.45839 |
| *CURS1* | GNT-2_SII | 1.88472 | 0.15455 | 26.97 | 0.10580 |
| *CURS1* | GNT-2_SIII | 1.08277 | 0.10151 | 27.26 | 0.17840 |
| *CURS1* | NDH-98 | 1.33614 | 0.14274 | 27.82 | 0.32412 |
| *CURS1* | NDH-98_SI | 2.05956 | 0.13529 | 27.21 | 0.13139 |
| *CURS1* | NDH-98_SII | 2.30762 | 0.25065 | 26.89 | 0.28665 |
| *CURS1* | NDH-98_SIII | 0.40444 | 0.04122 | 29.67 | 0.23404 |
| *CURS1* | Pratibha | 0.84297 | 0.12617 | 27.56 | 0.43559 |
| *CURS1* | Pratibha_SI | 0.50491 | 0.03533 | 29.11 | 0.22176 |
| *CURS1* | Pratibha_SII | 2.21519 | 0.10894 | 25.34 | 0.04443 |
| *CURS1* | Pratibha_SIII | 0.60089 | 0.12133 | 28.23 | 0.56833 |
| *CURS2* | GNT-2 | 0.74253 | 0.13052 | 29.75 | 0.27078 |
| *CURS2* | GNT-2_SI | 0.24630 | 0.05538 | 30.78 | 0.32725 |
| *CURS2* | GNT-2_SII | 1.05196 | 0.12848 | 29.73 | 0.05640 |
| *CURS2* | GNT-2_SIII | 1.67594 | 0.30308 | 28.55 | 0.19563 |
| *CURS2* | NDH-98 | 2.65075 | 0.30367 | 28.75 | 0.17820 |
| *CURS2* | NDH-98_SI | 2.90099 | 0.31039 | 28.64 | 0.07457 |
| *CURS2* | NDH-98_SII | 3.83366 | 0.62488 | 28.08 | 0.22190 |
| *CURS2* | NDH-98_SIII | 1.69248 | 0.23800 | 29.52 | 0.14969 |
| *CURS2* | Pratibha | 0.48516 | 0.09395 | 30.28 | 0.29074 |
| *CURS2* | Pratibha_SI | 0.57194 | 0.11020 | 30.86 | 0.34863 |
| *CURS2* | Pratibha_SII | 0.75786 | 0.07172 | 28.81 | 0.08241 |
| *CURS2* | Pratibha_SIII | 0.29559 | 0.06622 | 31.18 | 0.26836 |
| *CURS3* | GNT-2 | 1.29082 | 0.21770 | 28.17 | 0.20480 |
| *CURS3* | GNT-2_SI | 0.55497 | 0.15635 | 28.82 | 0.37028 |
| *CURS3* | GNT-2_SII | 2.87425 | 0.48254 | 27.50 | 0.15439 |
| *CURS3* | GNT-2_SIII | 1.35791 | 0.27257 | 28.07 | 0.17404 |
| *CURS3* | NDH-98 | 0.90504 | 0.11147 | 29.52 | 0.15925 |
| *CURS3* | NDH-98_SI | 0.90990 | 0.11566 | 29.53 | 0.11498 |
| *CURS3* | NDH-98_SII | 1.39024 | 0.23614 | 28.76 | 0.11825 |
| *CURS3* | NDH-98_SIII | 0.69745 | 0.13952 | 30.02 | 0.23742 |
| *CURS3* | Pratibha | 0.77540 | 0.13047 | 28.82 | 0.16965 |
| *CURS3* | Pratibha_SI | 1.09442 | 0.12548 | 29.14 | 0.17817 |
| *CURS3* | Pratibha_SII | 0.79133 | 0.09389 | 27.96 | 0.08718 |
| *CURS3* | Pratibha_SIII | 0.66702 | 0.15994 | 29.22 | 0.22143 |
| *DCS* | GNT-2 | 1.55348 | 0.16668 | 28.81 | 0.18124 |
| *DCS* | GNT-2_SI | 1.04619 | 0.23982 | 28.81 | 0.45402 |
| *DCS* | GNT-2_SII | 2.94024 | 0.26918 | 28.37 | 0.06366 |
| *DCS* | GNT-2_SIII | 1.12169 | 0.16185 | 29.25 | 0.21756 |
| *DCS* | NDH-98 | 0.66026 | 0.08463 | 30.87 | 0.26617 |
| *DCS* | NDH-98_SI | 1.12427 | 0.11104 | 30.12 | 0.13093 |
| *DCS* | NDH-98_SII | 0.87894 | 0.11703 | 30.32 | 0.22651 |
| *DCS* | NDH-98_SIII | 0.26896 | 0.03166 | 32.29 | 0.13688 |
| *DCS* | Pratibha | 1.03635 | 0.17744 | 29.30 | 0.32289 |
| *DCS* | Pratibha_SI | 1.13487 | 0.08990 | 29.98 | 0.17533 |
| *DCS* | Pratibha_SII | 1.55854 | 0.11633 | 27.88 | 0.07374 |
| *DCS* | Pratibha_SIII | 0.57999 | 0.15123 | 30.32 | 0.48128 |

*DCS*, diketide CoA synthase;

*CURS1*, *CURS2,* and *CURS3*, multiple curcumin synthases;

NDH-98, GNT-2 and Pratibha, turmeric cultivars;

SI, 4 months after planting (active vegetative stage);

SII, 5 months after planting (rhizome development stage);

SIII, 6 months after planting (maturity or senescence stage);

SEM, standard error of the mean;

Cq, threshold cycle

**Table S2. Gene specific and reference primers used in qPCR assay**

| Gene ID | Primer Sequence (5’ to 3’) | Primer Length | References | NCBI Reference sequence | Observed size in 2% FA gel |
| --- | --- | --- | --- | --- | --- |
| *DCS* (F) | GTGCTGTTCATCCTGGACGAG | 21bp | [1] | AB495006.1 | 92-94bp |
| *DCS* (R) | CAACAGCACGCCCCAGTCGA | 20bp |  |  |  |
| *CURS1* (F) | CATCATTGACGCCATCGAAGC | 21bp |  | AB495007.1 | 124-125bp |
| *CURS1* (R) | TCAGCTCATCCATCACGAAGTACAC | 25bp |  |  |  |
| *CURS2* (F) | TCGGGATCAAGGACTGGAACAAC | 23bp | [2] | AB506762.1 | 141-142bp |
| *CURS2* (R) | TGTTGCCGAACTCGGAGAAGAC | 22bp |  |  |  |
| *CURS3* (F) | TGGAGCCCTCCTTCGACGACC | 21bp |  | AB506763.1 | 95-97bp |
| *CURS3* (R) | CCCATTCCTTGATCGCCTTTTCC | 23bp |  |  |  |
| Actin (F) | GGATATGCTCTTCCTCATGCT | 21bp | [3] | CP002686.1  AK118354.1  AY087740.1 | 115-116bp |
| Actin (R) | TCTGCTGTGGTGGTGAATGA | 20bp |  |  |  |

bp, base pair(s);

F, forward primer;

R, reverse primer

FA, formaldehyde agarose gel

***References:***

1. Katsuyama Y, Kita T, Funa N, Horinouchi S (2009) Curcuminoid biosynthesis by two type III polyketide synthases in the herb *Curcuma longa*. J Biol Chem 284(17):11160-11170. doi:10.1074/jbc.M900070200.

2. Katsuyama Y, Kita T, Horinouchi S (2009) Identification and characterization of multiple curcumin synthases from the herb *Curcuma longa*. FEBS Lett 583(17):2799-2803. doi:10.1016/j.febslet.2009.07.029.

3. Chan SN, Abu Bakar N, Mahmood M, Ho CL, Mohamad Dzaki N, Shaharuddin NA (2016) Identification and expression profiling of a novel Kunitz trypsin inhibitor (KTI) gene from turmeric, *Curcuma longa*, by real-time quantitative PCR (RT-qPCR). Acta Physiologiae Plantarum 39(1):12. doi:10.1007/s11738-016-2311-7.

**Table S3. Duncan’s mean comparison of *DCS*, *CURS1*, *CURS2* and *CURS3* expressions among turmeric cultivars**

| **Cultivar** | ***Normalized gene expressions at p<0.05*** | | | |
| --- | --- | --- | --- | --- |
|  | ***DCS*** | ***CURS1*** | ***CURS2*** | ***CURS3*** |
| **GNT-2** | 1.38a | 0.62a | 0.91b | 1.35a |
| **Pratibha** | 0.81b | 0.62a | 0.41b | 0.7b |
| **NDH-98** | 0.54b | 0.9a | 2.14a | 0.74b |
| Means with the same letter (a,b) are not significantly different. | | | | |

*Note: Significant difference was found among cultivars for one or more gene expressions and only CURS2 has shown significant difference with >2-fold change. Thus, more precise association based test using robust regression was performed to study gene expression variation in association with curcuminoid biosynthesis in turmeric rhizome.*

**Annex 1. SAS codes for association-based test using ROBUSTREG procedure**

Following PROC ROBUSTREG statements were used to analyse the data in SAS studio:

/*

* Task code generated by SAS Studio 3.71

*/

ods noproctitle;

ods graphics / imagemap=on;

proc sort data=WORK.GENE out=Work.GeneDataSorted;

by Rank Cultivar;

run;

proc robustreg data=Work.GeneDataSorted plots=all;

class GrowthStage Genotype;

model Curcuminoid=GrowthStage Genotype DCS CURS1 CURS2 CURS3 / diagnostics leverage;

id Sample;

test CURS1 CURS2;

run;

proc robustreg data=Work.GeneDataSorted plots=all;

class GrowthStage Genotype;

model Curcumin=GrowthStage Genotype DCS CURS1 CURS2 CURS3 / diagnostics leverage;

id Sample;

test CURS1 CURS2;

run;

proc robustreg data=Work.GeneDataSorted plots=all;

class GrowthStage Genotype;

model Demethoxycurcumin=GrowthStage Genotype DCS CURS1 CURS2 CURS3 / diagnostics leverage;

id Sample;

test CURS1 CURS2;

run;

proc robustreg data=Work.GeneDataSorted plots=all;

class GrowthStage Genotype;

model Bisdemethoxycurcumin=GrowthStage Genotype DCS CURS1 CURS2 CURS3 / diagnostics leverage;

id Sample;

test CURS1 CURS2;

run;

///*********************************************************************///
