# Supplementary material for: Associating gene expressions with curcuminoid biosynthesis in turmeric
Source: J Genet Eng Biotechnol. 2020 Dec 14;18:83. doi: 10.1186/s43141-020-00101-2 (PMC7736439; doi:10.1186/s43141-020-00101-2)
Supplement: Supplementary file 2 — Additional file 2: Fig S1. Chromatograms and calibration curves for curcumin, demothoxycurcumin and bisdemethoxycurcumin standards using HPLC. Fig S2. Chromatograms showing individual curcuminoid contents measured using HPLC at three stages of growth of three turmeric cultivars. Fig S3. Melt peak curves and gel electrophoresis results for DCS, CURS1, CURS2, CURS3 and reference gene Actin. In melt peak curves, single peak of each gene specific product was observed in each sample. In gel image, N represents ‘NDH-98’; G represents, ‘GNT-2’; and P represents ‘Pratibha’ cultivars of turmeric under study. [file 43141_2020_101_MOESM2_ESM.docx]

Additional File 2.

Curcuminoids calibration curves

| 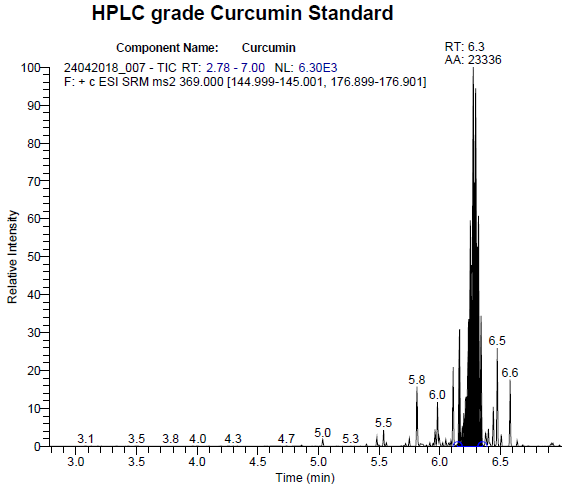 | 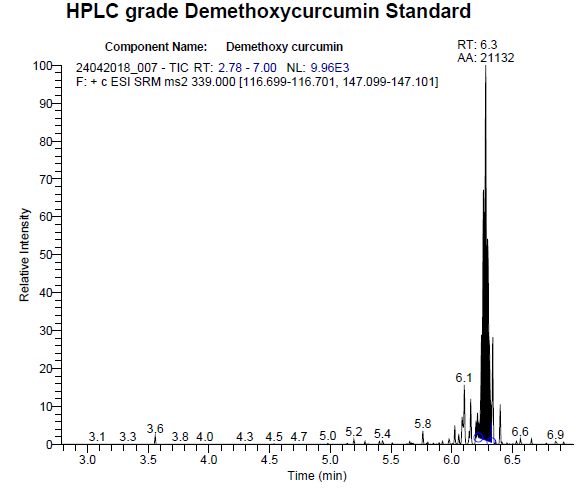 | 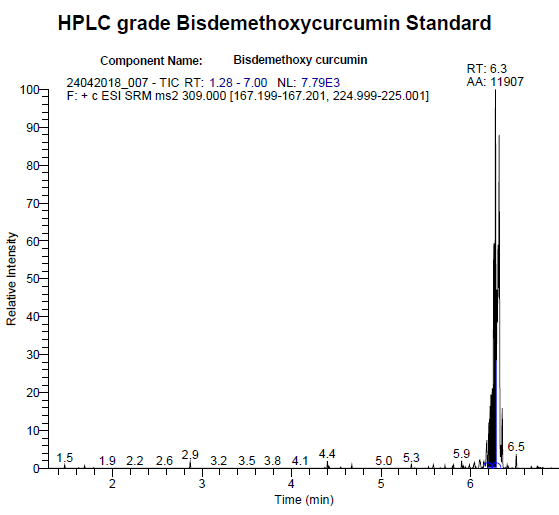 |
| --- | --- | --- |
| 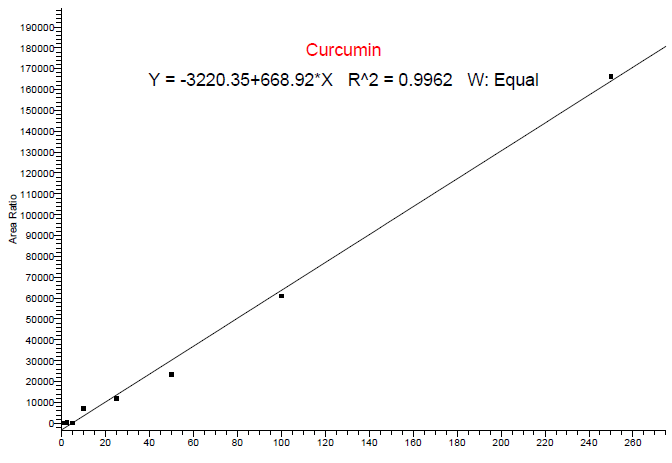 | 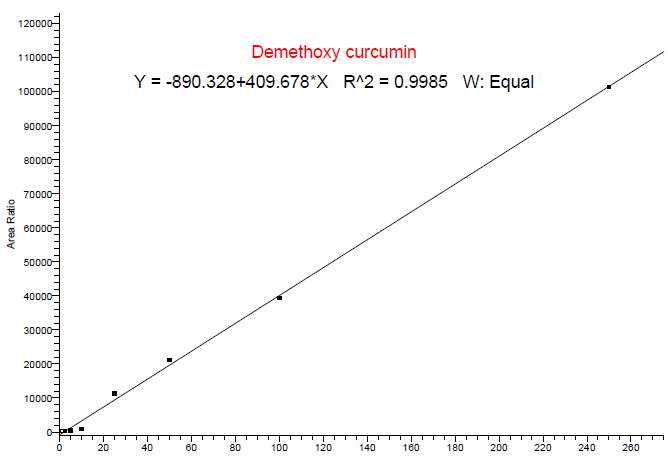 | 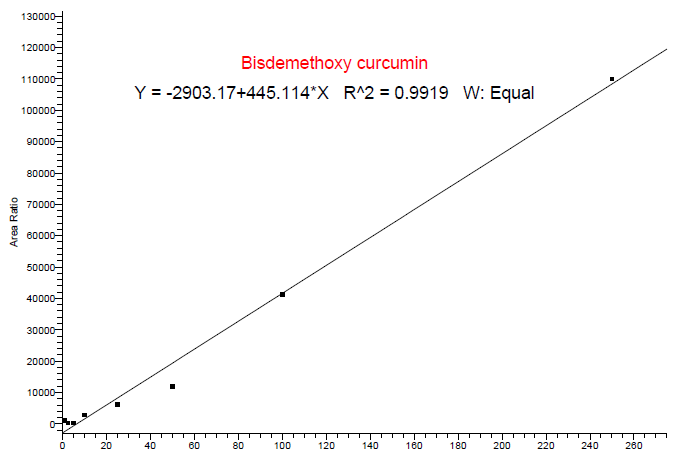 |
| **Fig S1. Chromatograms and calibration curves for curcumin, demothoxycurcumin and bisdemethoxycurcumin standards using HPLC.** | | |

Chromatograms showing quantification of curcuminoids in turmeric samples

| 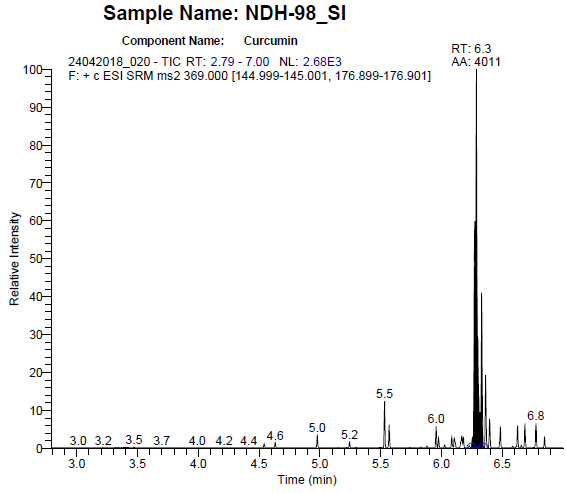 | 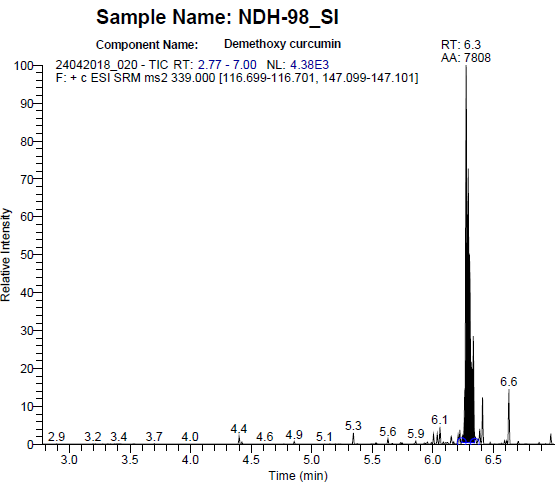 | 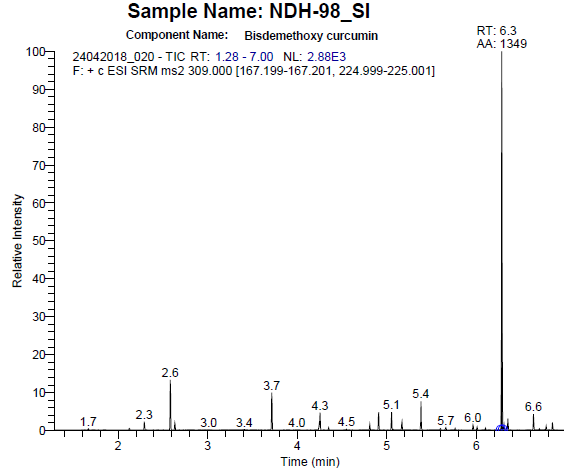 |
| --- | --- | --- |
| 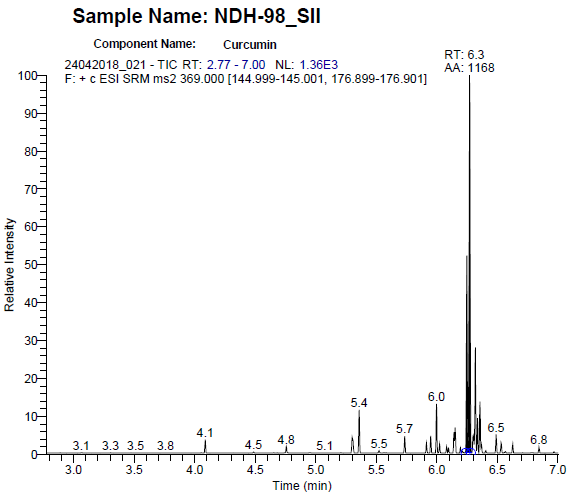 | 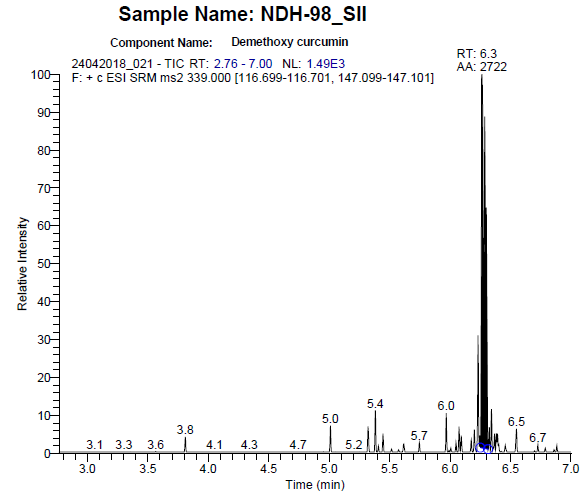 | 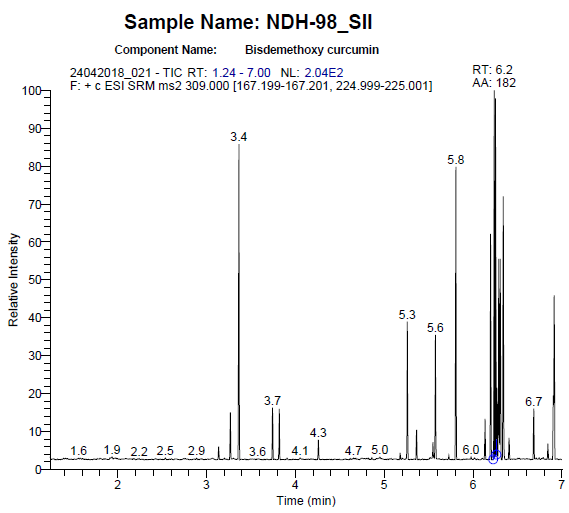 |
|  |  |  |
| 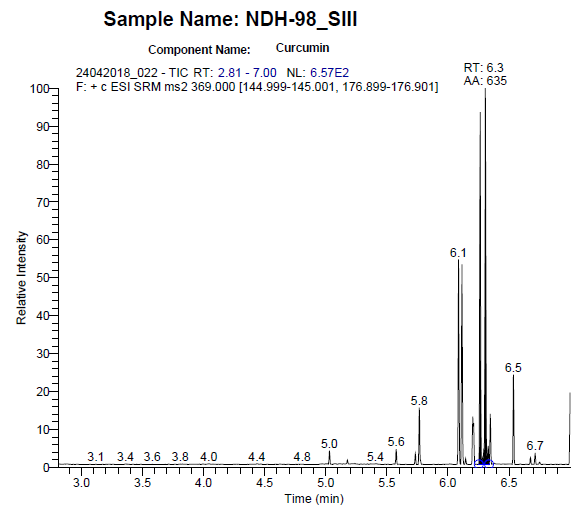 | 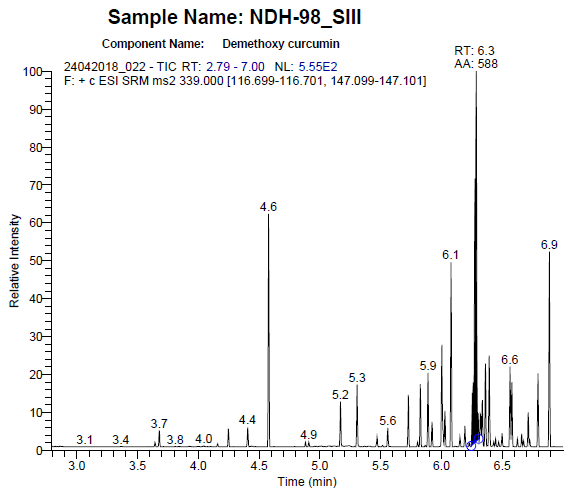 | 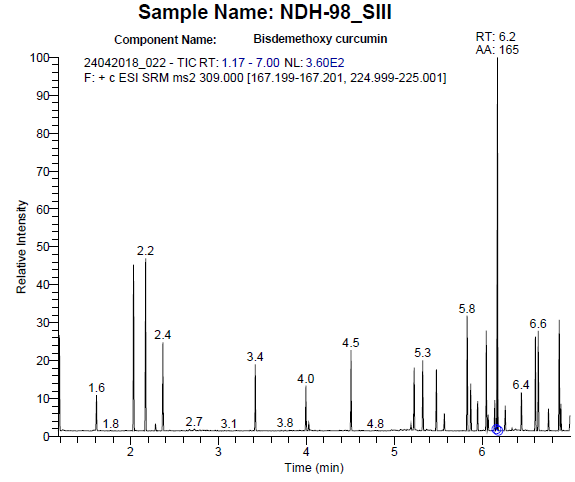 |
| 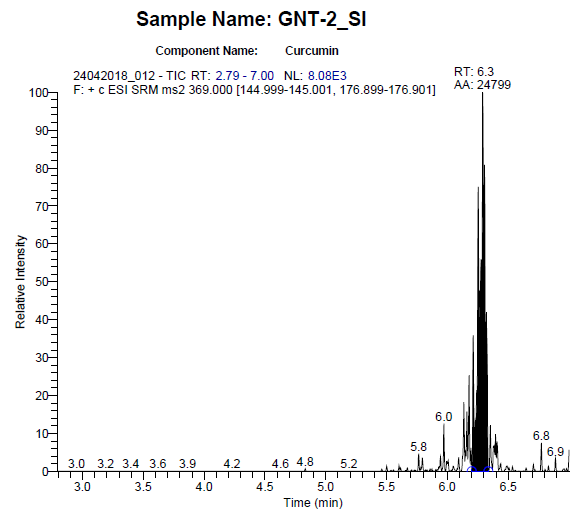 | 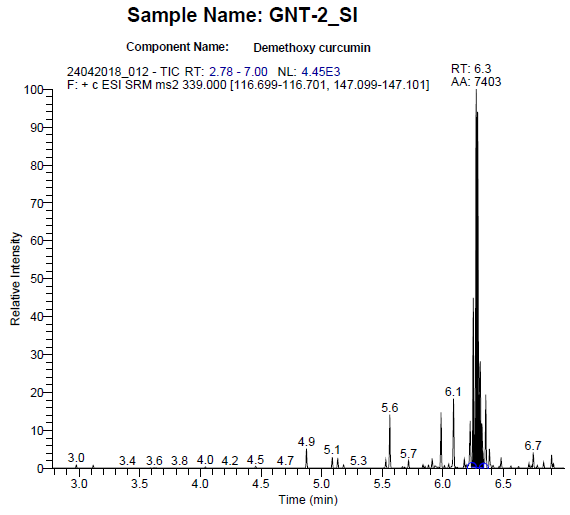 | 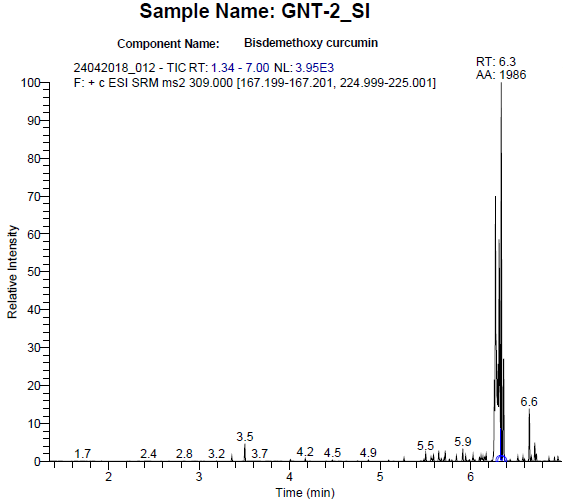 |
|  |  |  |
| 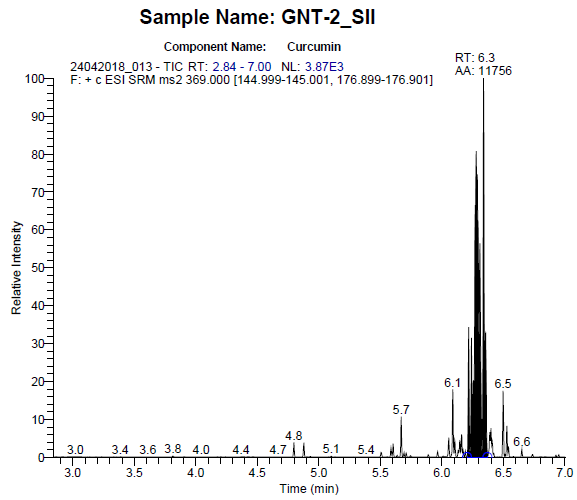 | 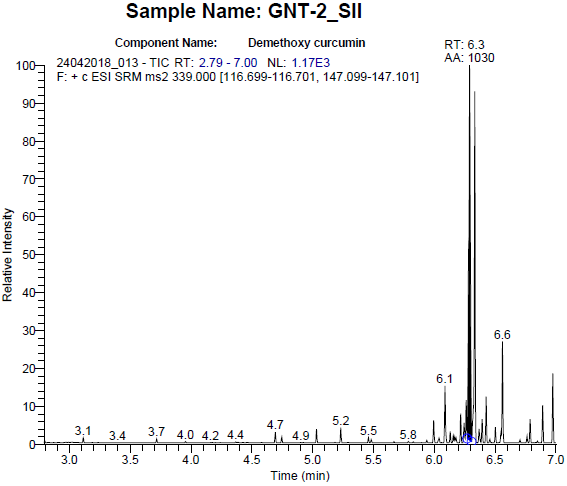 | 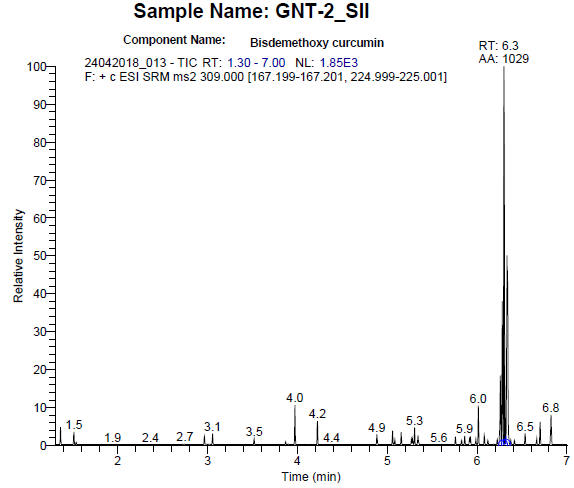 |
| 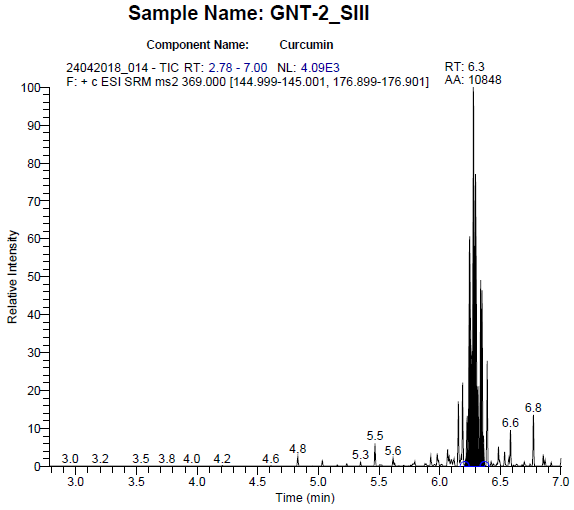 | 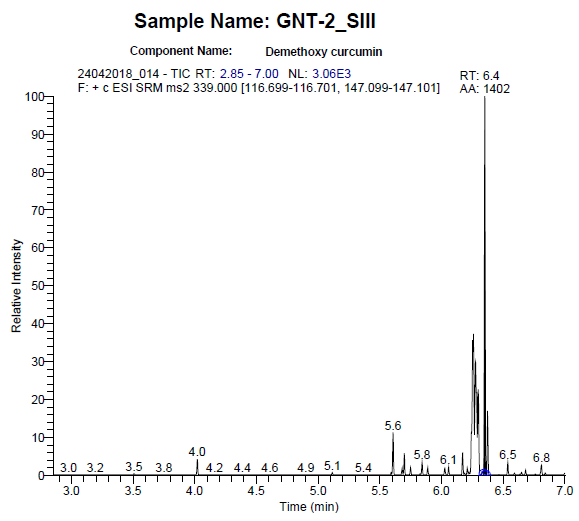 | 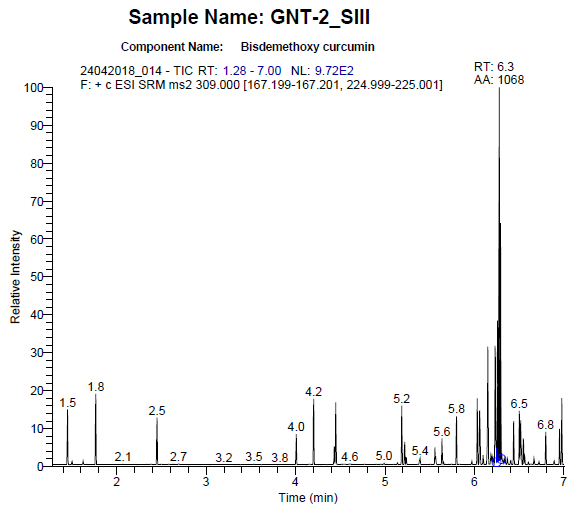 |
| 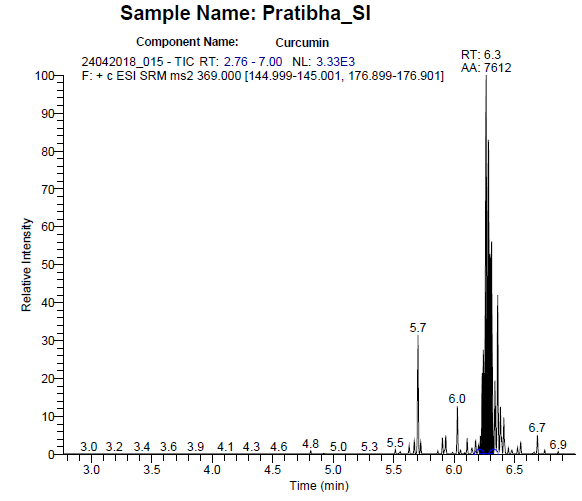 | 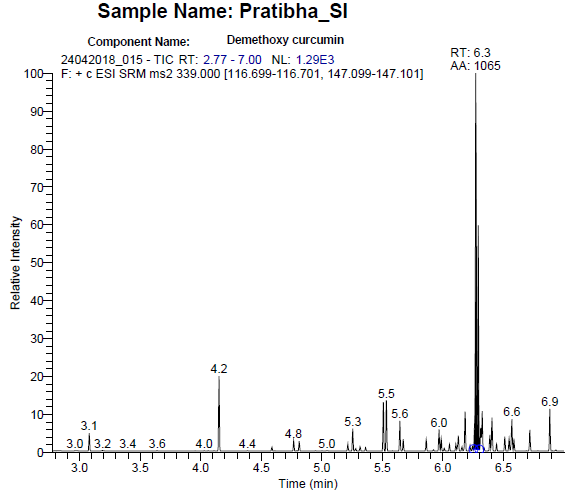 | 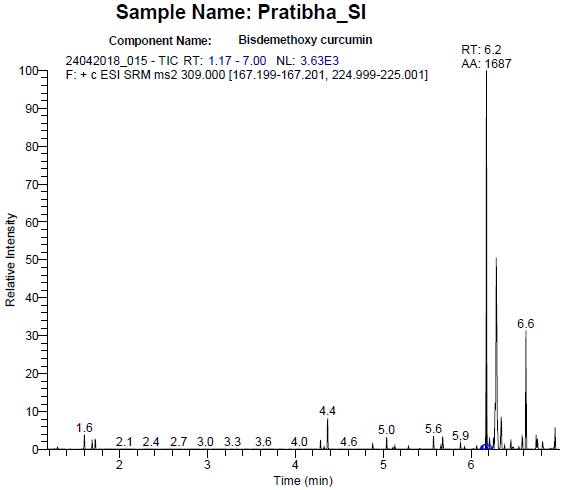 |
| 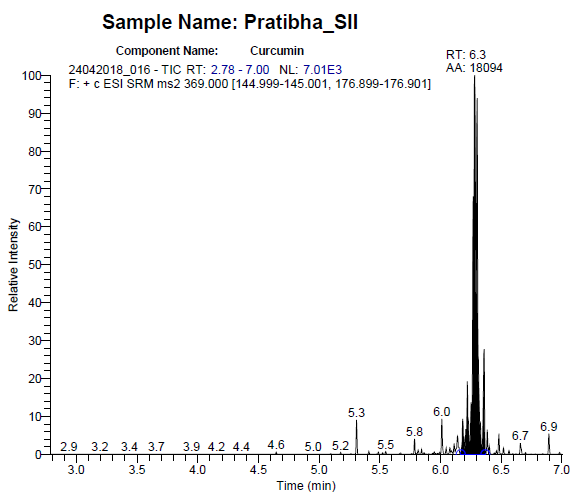 | 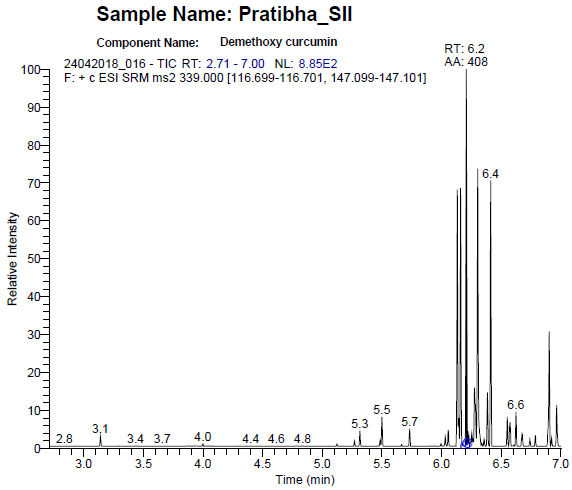 | 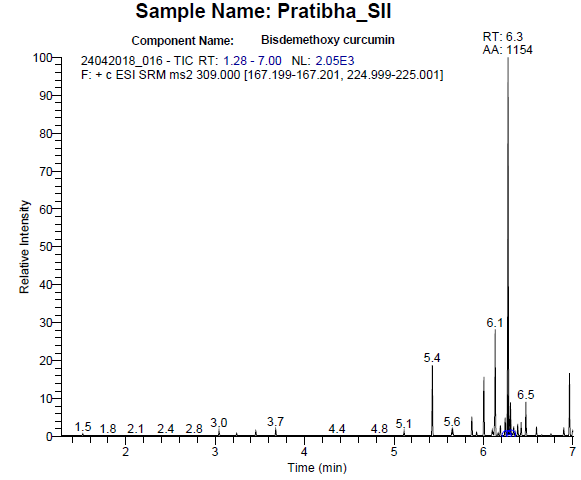 |
|  |  |  |
|  |  |  |
| 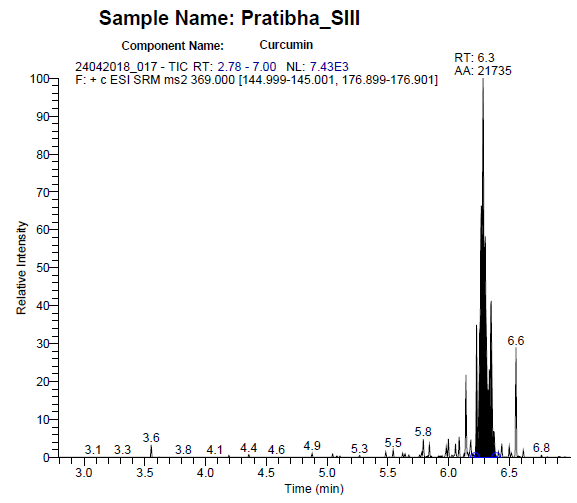 | 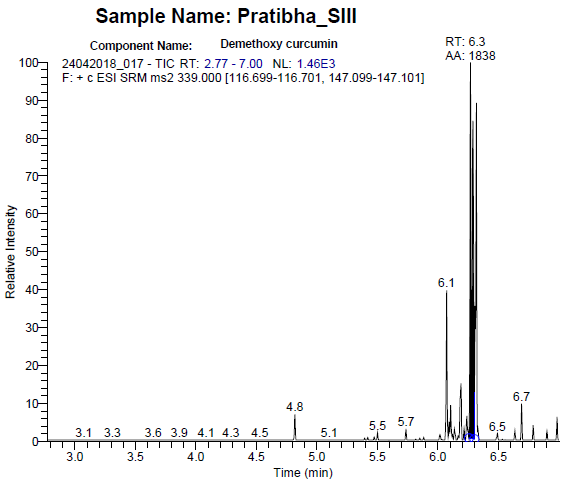 | 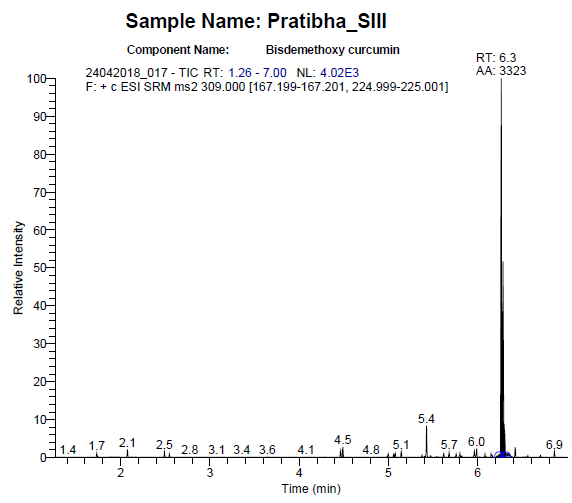 |
| **Fig S2. Chromatograms showing individual curcuminoid contents measured using HPLC at three stages of growth of three turmeric cultivars** | | |

Melt peaks (green graphs) and gel electrophoresis bands for *DCS* and *CURS*s in qPCR assay

| 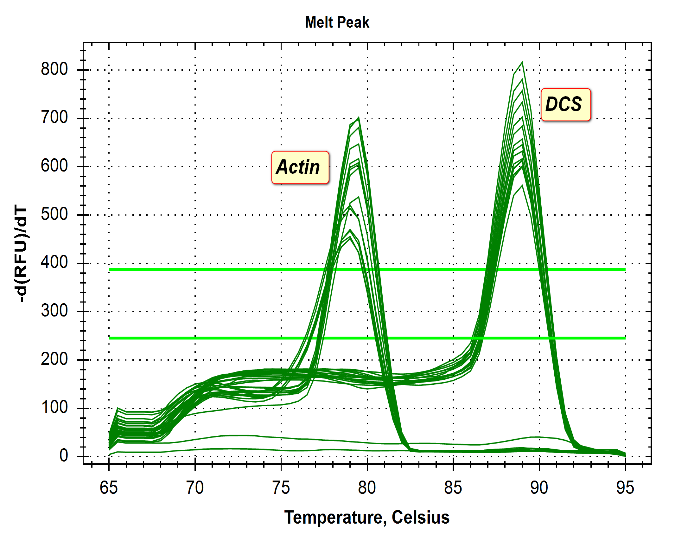 | 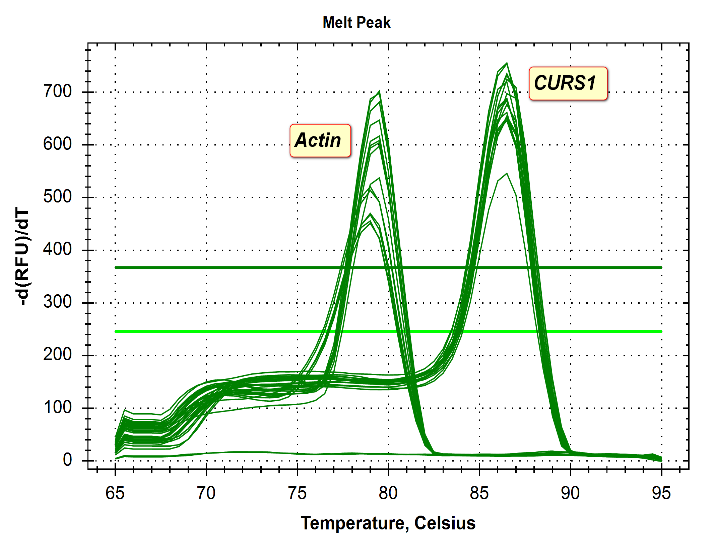 | 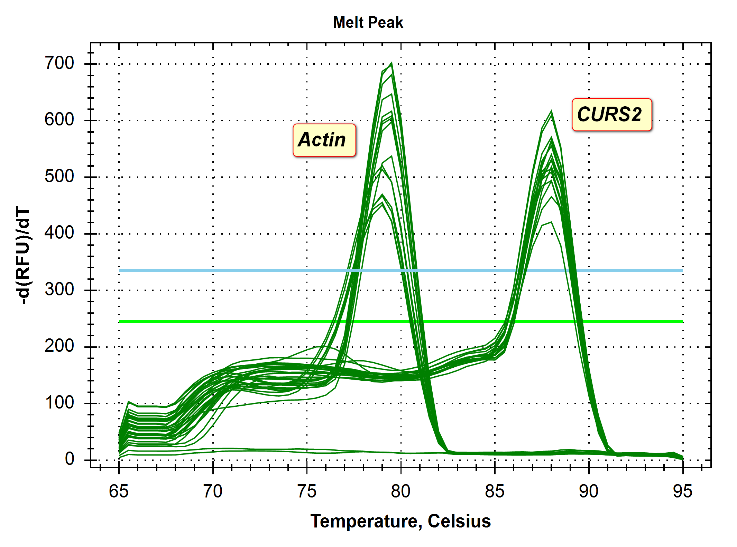 |
| --- | --- | --- |
| 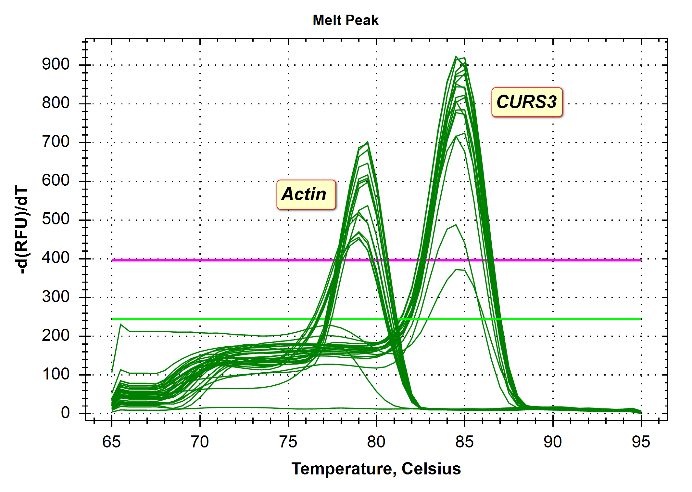 | 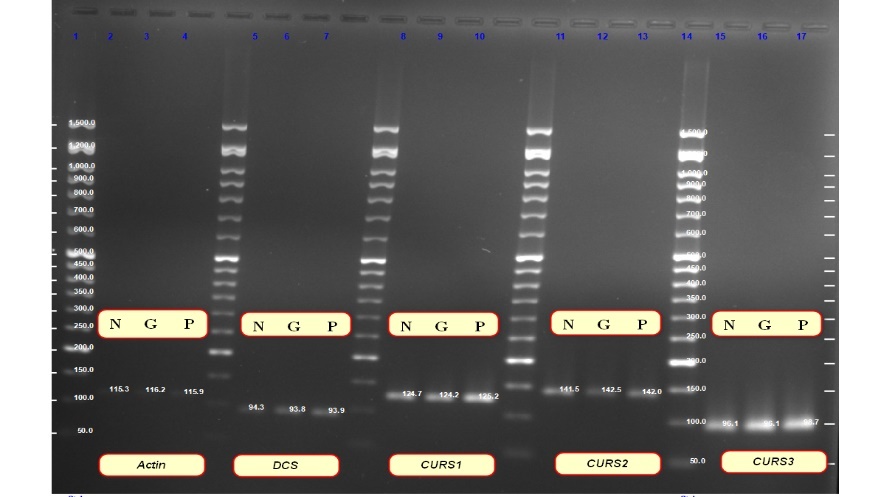 | |
| **Fig S3. Melt peak curves and gel electrophoresis results for *DCS*, *CURS1, CURS2, CURS3* and reference gene *Actin*.**  In melt peak curves, single peak of each gene specific product was observed in each sample. In gel image, N represents ‘NDH-98’; G represents, ‘GNT-2’; and P represents ‘Pratibha’ cultivars of turmeric under study. | | |
